# Gabapentin and cognitive impairment after traumatic brain injury: A multinational cohort of 49,925 patients

**DOI:** 10.1016/j.neurot.2026.e01041

**Published:** 2026-08-13

**Authors:** Isaac B. Thorman, Ariel Sacknovitz, Anjali Goyal, Austin G. Li, Aryan Malhotra, Eric Feldstein, Jude Al-Mufti, Patricia E. McGoldrick, Michael C. Schubert, Carrie R. Muh, Dennis J. Keselman, Mill Etienne, Steven M. Wolf, Tatyana R. Gitlevich, Merritt D. Kinon, Jon B. Rosenberg, Andrew Bauerschmidt, Saef Izzy, Stephan A. Mayer, Chirag D. Gandhi, Fawaz Al-Mufti

**Affiliations:** a School of Medicine, New York Medical College, Valhalla, NY, USA; b Department of Otolaryngology – Head and Neck Surgery, Johns Hopkins University School of Medicine, Baltimore, MD, USA; c Department of Neurosurgery, Westchester Medical Center, Valhalla, NY, USA; d Department of Pediatric Neurology, Boston Children’s Health Physicians, Hawthorne, NY, USA; e Department of Physical Medicine and Rehabilitation, Johns Hopkins University School of Medicine, Baltimore, MD, USA; f Department of Neurology, Westchester Medical Center, Valhalla, NY, USA; g Department of Neurology, Brigham and Women’s Hospital, Boston, MA, USA; h Department of Neurology, Mt. Sinai Hispital, New York, NY, USA; i Department of Surgery, Northwell Health, New Hyde Park, NY, USA

**Keywords:** Brain injury, Traumatic, Gabapentin, Cognitive impairment, Population-based, Head injury

## Abstract

Traumatic brain injury (TBI) is a major cause of morbidity and mortality, and cognitive impairment can be devastating among survivors. The objective was to assess an association between gabapentin and cognitive impairment after TBI. This retrospective cohort study used the multinational TriNetX Research Network (>150 million patients). Adults (≥18 years) with a first TBI and Glasgow Coma Scale (GCS) score recorded on the day of injury were included. Patients with known cognitive impairment or gabapentin exposure were excluded. The cohort (*n* = 49,925) was stratified into mild (GCS 13–15; *n* = 34,376), moderate (9–12; *n* = 4035), and severe (3–8; *n* = 12,845) TBI. The risk of cognitive impairment and mortality were assessed using Cox proportional hazard models adjusted for known predictors. Secondary analyses examined levetiracetam use (as seizure prophylaxis) and long-term medical and functional outcomes. Among 49,925 included patients w, 3.5% received gabapentin on the day of TBI. After adjustment, gabapentin was associated with a 22% lower risk of cognitive impairment in mild TBI (HR = 0.78; 95% CI, 0.62–0.98; *P* = .03) and a 46% lower risk of mortality in severe TBI (HR = 0.54; 95% CI, 0.40–0.72; *P* < .001). Levetiracetam showed no protective association with cognition. Long-term follow-up associated gabapentin use with lower mortality but higher rates of psychiatric/sleep diagnoses, reduced mobility, atrial fibrillation, and pulmonary embolism. Although causality cannot be inferred, these findings suggest gabapentin warrant prospective investigation as a candidate neuroprotective therapy.

## Introduction

Cognitive impairment is among the most devastating and persistent consequences of traumatic brain injury (TBI), affecting attention, executive function, and memory long after the acute injury [1,2]. More than 11 million Americans over the age of 40 are thought to currently live with disability in at least one functional domain after TBI [3,4]. These deficits persist for years, eroding quality of life and functional recovery while driving significant economic burden [5].

Despite decades of research, no pharmacologic intervention has reliably prevented the onset of chronic cognitive decline after TBI. Management of patients with TBI emphasizes seizure prophylaxis, various forms of therapy, symptom management, and supportive care [[6], [7], [8], [9]]. Patients who have suffered TBI are vulnerable to secondary injury processes such as excitotoxicity, dysautonomia, and neuroinflammation that unfold in the days and weeks following trauma [10]. Likewise, they may be at increased risk of developing seizures. Seizure prophylaxis with anti-seizure medications (ASMs) such as levetiracetam is controversial in certain patient populations but may demonstrate the possibility of neuroprotective care in acute neurotrauma [[11], [12], [13]].

To this effect, a recent population-level analysis of 52,000 patients with TBI found that levetiracetam reduced only the risk of early seizures in patients with only severe TBI, after adjusting for known predictors of post-traumatic epilepsy and poor neurological outcomes [14]. Gabapentin, originally developed as an antiseizure medication, has since become widely repurposed for neuropathic pain and agitation after TBI [[15], [16], [17], [18], [19]]. Beyond these symptomatic roles, we hypothesize that gabapentin may act as a disruptor in the field of TBI care. Gabapentin acts as a high-affinity ligand at the α2δ-1 and α2δ-2 receptors to block thrombospondin-1-mediated, astrocyte-induced excitatory synaptogenesis [[20], [21], [22]]. In neuron-glioma co-cultures, gabapentin exposure reduced individual spikes, spike bursts, and synchronized network bursts [23,24]; subsequent administration of gabapentin to human patients with glioblastoma resulted in a 35% reduction in the adjusted hazard of mortality and an absolute difference of 4–6 months of survival [25]. Extensive evidence from preclinical studies of spinal cord injuries [[26], [27], [28], [29], [30], [31], [32]], subsequently validated in human subjects [33,34], have shown improved motor recovery among gabapentinoid recipients. Other preclinical studies have shown neuroprotective effects in models of central nervous system trauma and ischemic injury [22,30,32,[35], [36], [37], [38], [39], [40]], while recent clinical observations link gabapentinoids to increased survival, improved functional outcomes, and fewer autonomic complications [18,22,37,41].

Building on our prior analysis [14], we leverage one of the largest multinational, federated clinical databases to examine whether gabapentin administration at the time of TBI is associated with reduced risk of durable cognitive impairment. By reframing gabapentin not as an ancillary pain medication, but as a potential neuroprotective therapy, we aim to challenge conventional management paradigms and explore whether this inexpensive, widely available drug could meaningfully alter the trajectory of recovery after TBI.

## Materials and Methods

### Ethics statement

We present a secondary analysis of existing data which does not involve intervention or interaction with human subjects and is de-identified per the standard defined in Section §164.514(a) of the HIPAA Privacy Rule. Only de-identified data was made accessible to the authors. Therefore, this study is exempt from Institutional Review Board/ethics committee approval and individual patient consent (i.e., IRB approval was not required); this was reaffirmed by the Johns Hopkins Medicine Institutional Review Board.

### Study design and data source

We conducted a retrospective, longitudinal cohort study using the TriNetX Research Network, a federated dataset which provided access to the deidentified electronic medical records (diagnoses, procedures, medications, laboratory values) of approximately 151 million patients from 107 healthcare organizations in multiple countries [42,43]. Of these healthcare organizations, 55 are academic, 44 are non-academic, and eight are an unknown type. Electronic medical record data were collected as part of routine clinical care in both inpatient and outpatient settings and are retrieved in a structured manner for demographics, diagnoses, procedures, medications, laboratory tests, and vital signs using accepted coding methodology [[44], [45], [46]]. Elements of narrative text from clinical documents were extracted by TriNetX using a proprietary Natural Language Processing program [45,47]. Data were collected for clinical purposes, reducing concerns regarding missing data; however, though TriNetX data has been published in more than 2500 studies, a ubiquitous limitation is that structured fields (including GCS and formal neuropsychological testing) are inconsistently coded [43]. Updates and new patients are added daily, so the number of patients present at the moment of each individual analysis may fluctuate.

### Study population

**Fig. 1:**
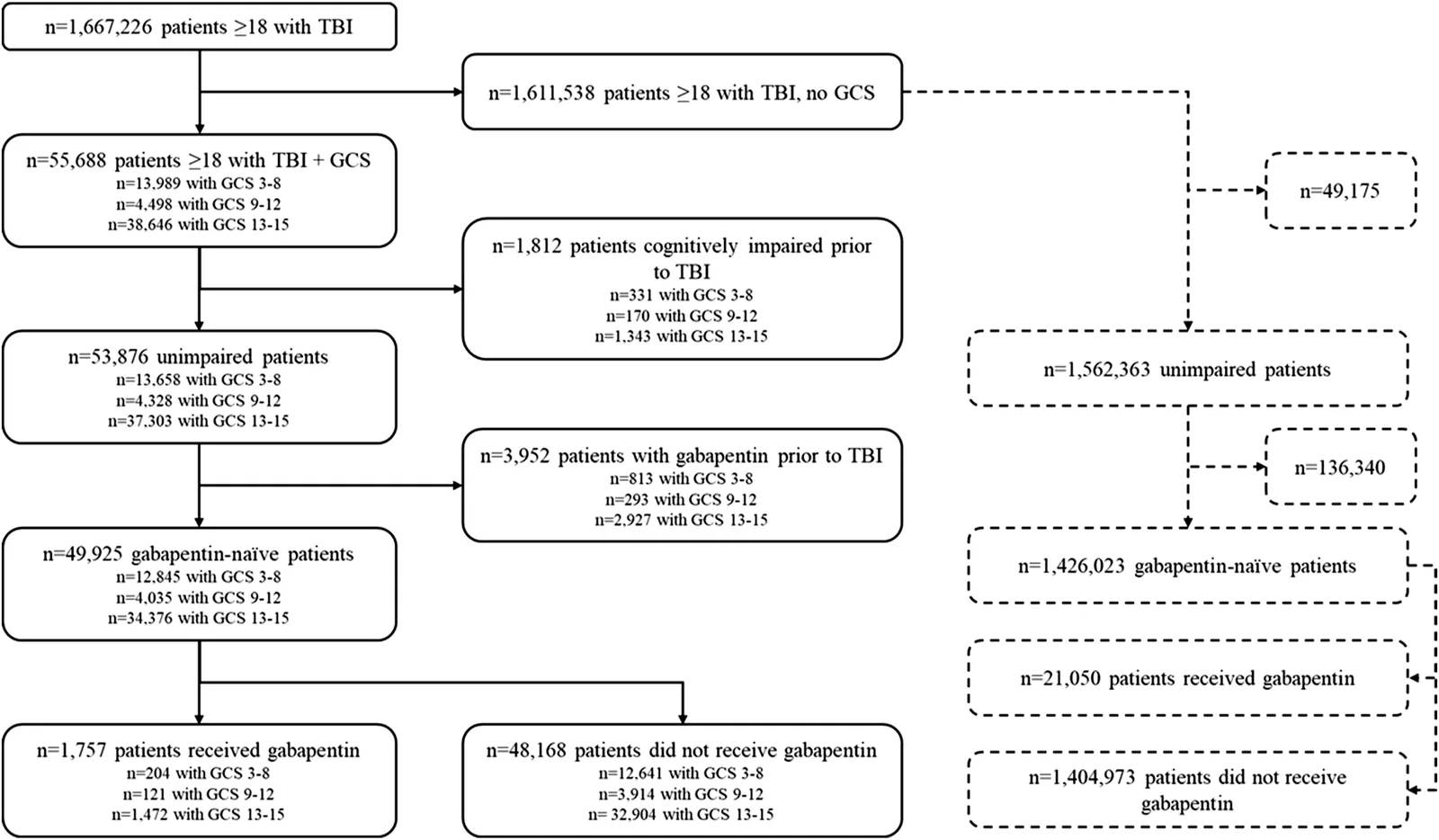
**Study Inclusion and Exclusion Flowchart.** Dashed lines indicate the pathway of patients who did not have Glasgow Coma Scale recorded on the day of TBI. This is included for reference only as these patients were not studied. Updates and new patients are added daily, so the number of patients present at the moment of each individual analysis may fluctuate; slight variations are present here. Summing patient counts by GCS categories differ slightly from the total number as 2.6% of patients appeared in multiple categories.

As in prior studies [14], we identified adult patients (≥18 years) with a diagnosis of TBI (ICD-10-CM code S06: intracranial injury) since 2005 (Fig. 1), and we excluded patients with known gabapentin exposure or durable cognitive impairment prior to their TBI. Our analysis focused on patients with Glasgow Coma Scale (GCS) recorded on the day of their TBI, and we grouped these patients into mild TBI (GCS 13–15), moderate TBI (GCS 9–12), and severe TBI (GCS 3–8) based on ICD-10 codes (R40.241, R40.242, and R40.243, respectively). GCS has been used in more than 80% of randomized controlled trials of TBI as the primary determinant of severity [48]. We found that 2.6% of patients had more than one GCS category reported on the day of their TBI; as we could not determine which category was recorded first, these patients were included in each recorded category. We chose to include these patients across categories of severity as we believe these patients represent an important category of TBI presentations: most likely, these patients acutely decompensated shortly after initiation of medical care, and it is critical that our findings generalize to this population.

### Primary outcomes

The primary outcomes were durable cognitive impairment and all-cause mortality within two years of TBI. We defined durable cognitive impairment as any of the following: vascular dementia (F01), dementia in other diseases (F02), unspecified dementia (F03), Alzheimer dementia (G30), other neurodegenerative disorders (G31, includes frontotemporal dementia, mild cognitive impairment, dementia with Lewy bodies), and other degenerative disorders of the nervous system (G32). These codes have been used in prior publications and will be grouped under a more cohesive paradigm in the ICD-11 [16,44,49,50]. Mortality data is collected by TriNetX from healthcare organization death records, billing codes, the Social Security Administration Master Death File, private obituaries, and private claims.

### Exposure definition

The exposure was gabapentin prescription on the day of TBI as determined by RxNorm code 25480. Gabapentin was prescribed by oral (71%) or unspecified (29%) route for all patients (i.e., no intravenous or topical prescriptions were observed). All prescriptions were assumed to have been taken as directed. Initial gabapentin doses of 100 mg and 300 mg were studied in the dose-response analyses as these were most commonly observed.

### Statistical analyses

Statistical analyses were performed using TriNetX’s Advanced Analytics Platform, using R version 3.4.4 (R Foundation for Statistical Computing, Vienna, Austria), and Python version 3.6.5 (Python Software Foundation, Centrum voor Wiskunde en Informatica, Amsterdam, The Netherlands). Statistical significance was established at *α* < 0.05. The Advanced Analytics Platform does not return patient counts from queries with 1–10 patients, reporting all such values as “1–10.”

We estimated the prevalence of cognitive impairment and in the two years following TBI stratified by severity and gabapentin exposure. We compared the medical comorbidities of patients by gabapentin status using the diagnoses listed in the Charlson Comorbidity Index. Comorbidities were assessed up to and including the day of TBI with the exception of cerebrovascular conditions, which were assessed up to the day prior to TBI as they were potential complications of TBIs and the time resolution of the database did not allow for the determination of whether they were a pre-existing condition or a consequence. Comorbidities were ascertained from the diagnoses present in the electronic medical record, and TriNetX tracks patients across encounters in different healthcare organizations using unique patient identifiers which are not made available to us. Therefore, while some patients may have been able to provide missing medical history, it is possible that patients with more severe injuries were unable to update their records at the time of their injury. We also compared the presenting factors of patients by gabapentin status on the day of TBI, including demographics (age and sex), neurologic and psychiatric diagnoses, injury types, signs and symptoms, diagnostic and laboratory tests, and medical and procedural management, in the manner previously published [14]. Medical diagnoses were ascertained by ICD-10 codes, laboratory and diagnostic tests and procedural interventions were ascertained by Current Procedural Terminology (CPT) codes, and medications administered were ascertained by Veterans Affairs and RxNorm codes.

We used multivariate Cox proportional hazard models to assess the efficacy of gabapentin in preventing durable cognitive impairment in the two years following TBI. The proportional hazard assumption was tested by the scaled Schoenfeld residual test and was not violated in any reported model [51]. Models were adjusted for age, sex, type of intracranial pathology, location of focal traumatic injury, injuries to the head and neck, procedural interventions, and other parameters, in line with Guidelines for the Management of Severe Traumatic Brain Injury, Fourth Edition from the Brain Trauma Foundation. Covariates were all assessed on the day of TBI. GCS categories were included as covariates in models which included patients across all three severity strata. Patients with GCS 9–12 were not studied as such in their own set of models because there were considerably fewer of them (*n* = 4035) compared to GCS 13–15 (*n* = 34,376) and GCS 3–8 (*n* = 12,845), and this patient count was not sufficient to return results from the fully adjusted multivariable Cox proportional hazard model. Models were performed as follows:

● Supplemental Table 3 - Supplemental Table 5: risk of durable cognitive impairment overall (Supplemental Table 3), in mild TBI (Supplemental Table 4), and severe TBI (Supplemental Table 5)
● Supplemental Table 6 - Supplemental Table 7: risk of durable cognitive impairment in mild TBI at gabapentin doses of 100 mg (Supplemental Table 6) and 300 mg (Supplemental Table 7)
● Supplemental Table 8 - Supplemental Table 10: risk of mortality overall (Supplemental Table 8), in mild TBI (Supplemental Table 9), and in severe TBI (Supplemental Table 10)
● Supplemental Table 11: risk of durable cognitive impairment overall, additionally controlling for levetiracetam
● Supplemental Table 12 - Supplemental Table 14: risk of durable cognitive impairment overall (Supplemental Table 12), in mild TBI (Supplemental Table 13), and in severe TBI (Supplemental Table 14), additionally controlling for levetiracetam, seizures, and epilepsy
● Supplemental Table 15 - Supplemental Table 17: risk of mortality overall (Supplemental Table 15), in mild TBI (Supplemental Table 16), and in severe TBI (Supplemental Table 17), additionally controlling for levetiracetam, seizures, and epilepsy

As there were insufficient patients to study moderate TBI as GCS 9–12, we created a hybrid definition of moderate TBI for a sensitivity analysis (criteria listed with full explanation in Supplemental Table 17). Briefly, we included patients with GCS 9–12 and patients with responses that were below optimal but were not completely unresponsive in each category, and we excluded patients meeting criteria from the National Inpatient Sample – Subarachnoid Hemorrhage Severity Scale as these patients likely presented with severe TBI. We removed these criteria as covariates in the models for both durable cognitive impairment (Supplemental Table 19) and mortality (Supplemental Table 20).

We assessed for possible long-term adverse outcomes which may have followed gabapentin administration. We used 1:1 propensity score matching on age categories, sex, and GCS categories. A nearest-neighbor method was utilized with a caliper of 0.1 pooled standard deviations and without replacement [47]. Propensity score matching was used here as absolute measures of adverse outcomes were desired, while Cox proportional hazard models were used to assess for associations between gabapentin, cognitive impairment, and mortality as relative measures were desired. Outcomes were assessed within five years of TBI. For each outcome, we excluded patients with the outcome of interest on or before the day of TBI. Possible adverse events were selected based on prior studies of adverse events in ASMs and causes of morbidity and mortality in the setting of neurological trauma [4,14,47,[52], [53], [54], [55]].

## Results

### Prevalence of durable cognitive impairment and mortality following TBI

Among 49,925 included adult patients with a TBI and recorded GCS, 69% were mild TBIs, 8% were moderate TBIs, and 26% were severe TBIs (Fig. 1). The prevalence of durable cognitive impairment in the first two years was 6.6% in mild TBI, 11.6% in moderate TBI, and 7.1% in severe TBI. A potential competing risk of death was observed, as has been reported in numerous studies of TBI and other relevant disease processes [44,[56], [57], [58], [59], [60], [61], [62], [63], [64], [65]]. Over the same period, mortality rose sharply with severity: 4.8% in mild TBI, 15.4% in moderate TBI, and 39.3% in severe TBI.

### Properties of patients receiving gabapentin

On the day of TBI, 3.5% of patients received gabapentin, including 4.3% of patients with mild TBI, 3.0% of patients with moderate TBI, and 1.6% of patients with severe TBI. Patients receiving gabapentin had more risk factors for cognitive impairment than patients not receiving gabapentin. Gabapentin recipients were older at the time of TBI, experienced more prevalent acute pain due to trauma, reported more significant psychiatric comorbidities, and received more opioid analgesics (Table 1). There were no clinically significant differences in past medical history (Supplemental Table 1), but gabapentin recipients were more symptomatic and received many more pharmacological interventions than nonrecipients (Supplemental Table 2). Of note, levetiracetam was administered to an equal proportion of patients receiving and not receiving gabapentin (both 32.5%).

**Table 1:** Key population baseline characteristics.

Characteristic name	Gabapentin		No gabapentin		*p*-value
	Patients	Frequency	Patients	Frequency	
Age at TBI (mean ± SD years old)	1733	51.6 ± 18.8	47,287	48.4 ± 20.3	**<0.0001**
Sex					
Male	1061	61.2%	30,467	64.4%	**0.006**
Female	525	30.3%	14,633	30.9%	0.565
Medical comorbidities prior to the day of TBI					
Diabetes mellitus	282	16.1%	6206	12.9%	0.193
Chronic lower respiratory diseases	246	14.0%	6394	13.3%	**0.027**
Chronic ischemic heart disease	209	11.9%	5036	10.5%	0.056
Chronic kidney disease (CKD)	94	5.4%	2746	5.7%	0.060
Presenting symptoms and diagnoses on the day of TBI					
Substance use disorders	721	41.6%	16,652	35.2%	**<0.001**
Acute pain due to trauma	578	33.4%	6113	12.9%	**<0.001**
Nontraumatic subarachnoid hemorrhage	317	18.3%	8081	17.1%	0.192
Retention of urine	191	11.0%	2745	5.8%	**<0.001**
Aphagia and dysphagia	170	9.8%	4536	9.6%	0.763
Altered mental status, unspecified	102	5.9%	3846	8.1%	**<0.001**
Nontraumatic intracerebral hemorrhage	89	5.1%	3869	8.2%	**<0.001**
Nausea and vomiting	54	3.1%	1930	4.1%	**0.045**
Patient management on the day of TBI					
Opioid analgesics	1570	90.6%	28,194	59.6%	**<0.001**
CT, head or brain	1143	66.0%	33,458	70.8%	**<0.001**
Levetiracetam	564	32.5%	15,390	32.5%	0.999
Benzodiazepines	550	31.7%	10,863	23.0%	**<0.001**
CTA Head with contrast	254	14.7%	6638	14.0%	0.467
Surgery on the musculoskeletal system	221	12.8%	2658	5.6%	**<0.001**

**Bold** highlights significance at 95% confidence level. CT - computed tomography; CTA - CT angiography.

### Effects of gabapentin on cognitive impairment and mortality following TBI

Before adjustment, cognitive impairment within two years of TBI developed in 6.3% of patients receiving gabapentin and 7.0% of patients not receiving gabapentin (*P* = .21). When stratified by severity, prevalence was slightly lower in gabapentin recipients after mild (5.7% vs. 6.7%, *P* = .13) and moderate TBI (9.0% vs. 11.6%, *P* = .38), but slightly higher after severe TBI (8.4% vs. 7.1%, *P* = .47); none of these differences reached significance.

Mortality showed a different pattern. Prior to adjustment, gabapentin users had numerically lower two-year mortality across all severities, with the most striking difference in severe TBI (23.3% vs. 39.4%, *P* < .0001). Smaller, non-significant differences were observed in mild (4.6% vs. 4.8%, *P* = .74) and moderate TBI (11.4% vs. 15.8%, *P* = .19).

After multivariable adjustment including Glasgow Coma Scale (GCS) as a covariate, gabapentin was not associated with cognitive protection in the overall cohort (Fig. 1, Supplemental Table 3). However, stratified analyses demonstrated that patients with mild TBI who received gabapentin had a 22% reduced risk of durable cognitive impairment compared with non-users (HR 0.78, 95% CI 0.62–0.98, *P* = .03; Supplemental Table 4). No association was observed in severe TBI (HR 1.13, 95% CI 0.70–1.84, *P* = .61; Supplemental Table 5).

In contrast, gabapentin was consistently protective against mortality. After full adjustment, gabapentin use was associated with a 33% reduction in overall two-year mortality (HR 0.67, 95% CI 0.56–0.80, *P* < .0001; Supplemental Table 8). The effect was most pronounced in severe TBI, where mortality was reduced by nearly half (HR 0.54, 95% CI 0.40–0.72, *P* < .0001; Supplemental Table 10). No significant mortality benefit was observed in patients with mild TBI (HR 0.91, 95% CI 0.70–1.17, *P* = .44; Supplemental Table 9).

### Dose-response analysis in mild TBI

Among patients with mild TBI, gabapentin was most commonly initiated at doses of 100 mg (*n* = 332) or 300 mg (*n* = 582). Before adjustment, there were no differences in cognitive impairment (5.8% vs. 5.5%, *P* = .87) or mortality (6.1% vs. 4.0%, *P* = .15) between the two dosing groups.

After adjustment, however, dose-specific effects emerged. Patients prescribed the 100 mg dose experienced a significant reduction in cognitive impairment compared with those not treated (HR 0.58, 95% CI 0.37–0.92, *P* = .02; Supplemental Table 6). In contrast, the 300 mg dose showed no protective association (HR 0.93, 95% CI 0.65–1.32, *P* = .67; Supplemental Table 7).

### Effectiveness of levetiracetam in prevention of cognitive impairment and mortality

**Fig. 2:**
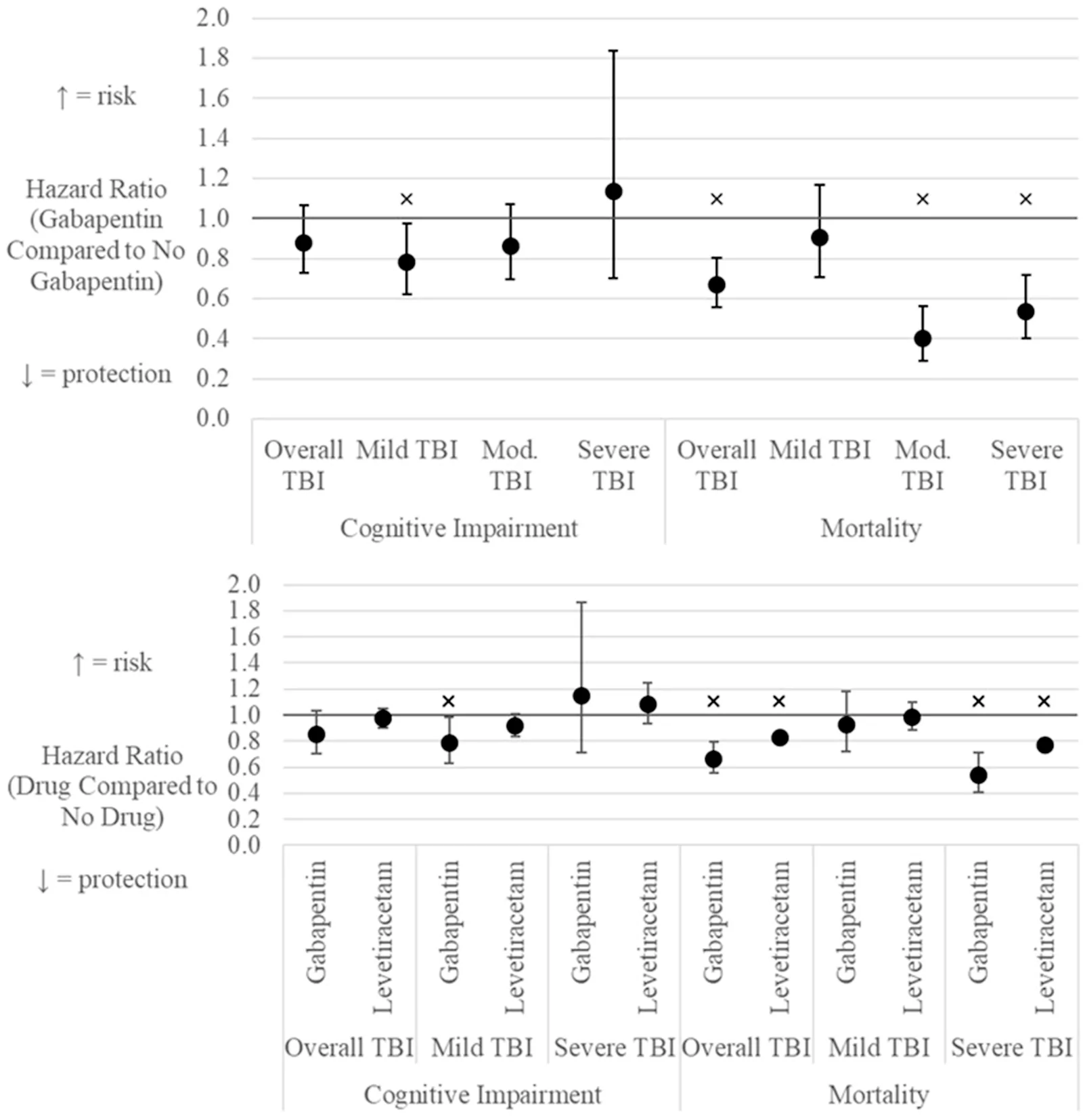
**Hazards of Cognitive Impairment and Mortality by Gabapentin Status.** Hazard of cognitive impairment and mortality associated with gabapentin (**Top**) and associated with both gabapentin and levetiracetam (**Bottom**). × indicates significance at 95% confidence level.

Levetiracetam was administered in 32.5% of both gabapentin-treated and untreated patients. When included as a covariate, levetiracetam showed no independent association with cognitive impairment (HR 0.997, 95% CI 0.92–1.07, *P* = .93; Supplemental Table 11), even after accounting for seizures and epilepsy (Supplemental Table 12) (Fig. 2).

Stratified analyses reaffirmed gabapentin’s unique signal. In mild TBI, gabapentin remained protective against cognitive impairment (HR 0.78, 95% CI 0.63–0.98, *P* = .03; Supplemental Table 13), whereas levetiracetam showed only a nonsignificant trend (HR 0.92, 95% CI 0.84–1.01, *P* = .09). In severe TBI, neither gabapentin (HR 1.15, 95% CI 0.71–1.87, *P* = .57) nor levetiracetam (HR 1.08, 95% CI 0.94–1.25, *P* = .28) was associated with cognitive outcomes (Supplemental Table 14). Across all models, epilepsy diagnosed on the day of TBI consistently predicted increased risk of cognitive impairment.

Both drugs, however, were associated with less mortality. Gabapentin and levetiracetam each were associated with reduced overall mortality (Supplemental Table 15) and mortality in severe TBI (Supplemental Table 16), though no mortality benefits were observed in mild TBI (Supplemental Table 17).

### Adverse effects and long-term associations of gabapentin

In a 1:1 propensity-matched analysis adjusting for age, sex, and GCS severity, patients receiving gabapentin had lower five-year mortality compared with matched controls (8.4% vs. 11.3%, *P* = .005). Long-term outcomes revealed a mixed profile of associations (Table 2). Patients treated with gabapentin had higher subsequent rates of psychiatric and sleep-related disorders, including substance use, anxiety, depression, and insomnia. They also more frequently developed worsened functional outcomes, such as reduced mobility, and select cardiovascular complications, such as atrial fibrillation and pulmonary embolism.

**Table 2:** Five-year adverse events associated with gabapentin.

Outcome	Gabapentin	No gabapentin	Risk difference	*p*-value
Mortality	8.4%	11.2%	−2.8%	0.007
Neurologic outcomes				
Headache and migraine	16.4%	17.9%	−1.5%	0.255
**Sleep disorders**	**14.5%**	**9.9%**	**4.6%**	**<0.001**
Impaired memory/awareness	10.9%	11.7%	−0.9%	0.470
**Malaise and fatigue**	**9.0%**	**7.0%**	**2.1%**	**0.031**
Epilepsy	6.4%	5.7%	0.7%	0.365
Dizziness and giddiness	6.3%	6.5%	−0.2%	0.772
Acute ischemic stroke	5.1%	5.0%	0.2%	0.838
Visual disturbances and blindness	4.9%	4.4%	0.5%	0.463
Non-visual cranial nerve defects	1.5%	1.5%	0.1%	0.886
Vestibular dysfunction	1.3%	1.1%	0.2%	0.645
Dystonia	1.3%	1.1%	0.1%	0.723
Transient ischemic attack	0.9%	1.1%	−0.2%	0.484
Psychiatric outcomes				
**Substance use disorders**	**40.3%**	**34.9%**	**5.4%**	**0.002**
**Anxiety disorders**	**21.9%**	**17.2%**	**4.8%**	**0.001**
**Depressive disorders**	**17.8%**	**14.5%**	**3.3%**	**0.011**
Delusional disorders	2.3%	2.4%	−0.1%	0.895
Cardiovascular and respiratory outcomes				
Mechanical cardiac support	18.8%	19.8%	−1.0%	0.443
Mechanical ventilation	15.9%	17.7%	−1.7%	0.177
**Atrial fibrillation and flutter**	**10.2%**	**8.1%**	**2.1%**	**0.033**
Influenza and pneumonia	9.6%	9.6%	0.0%	0.975
**Pulmonary embolism**	**3.3%**	**1.9%**	**1.4%**	**0.011**
Acute myocardial infarction	3.1%	3.8%	−0.7%	0.280
Deep vein thrombosis	2.3%	1.8%	0.5%	0.344
**Cardiac arrest**	**1.6%**	**3.2%**	**−1.5%**	**0.004**
Acute respiratory distress syndrome	1.6%	1.3%	0.3%	0.477
Cardiogenic shock	0.8%	1.1%	−0.3%	0.362
Respiratory arrest	0.6%	0.6%	0.0%	0.998
Metabolic, hepatic, and renal outcomes				
**Hyponatremia**	**39.5%**	**34.5%**	**5.1%**	**0.003**
Metabolic disorders	13.0%	11.8%	1.2%	0.457
Acute kidney failure	12.1%	12.0%	0.1%	0.895
Diseases of liver	8.0%	8.1%	−0.1%	0.906
Overweight and obesity	5.3%	4.5%	0.9%	0.262
**Hyperkalemia**	**4.7%**	**3.1%**	**1.6%**	**0.014**
**Diabetes mellitus**	**2.4%**	**3.7%**	**−1.3%**	**0.045**
Urolithiasis	1.6%	1.7%	−0.1%	0.850
Elevated liver transaminases	1.0%	1.2%	−0.2%	0.520
Urinary incontinence	0.9%	1.1%	−0.2%	0.486
Functional and miscellaneous outcomes				
Falls	33.6%	34.8%	−1.2%	0.468
**Reduced mobility**	**12.9%**	**6.1%**	**6.7%**	**<0.001**
Fever	4.5%	4.3%	0.2%	0.808
Thrombocytopenia	2.4%	2.2%	0.2%	0.728
Rash	2.2%	1.4%	0.7%	0.104
Sexual dysfunction	1.4%	1.0%	0.4%	0.256
Dependence on wheelchair	1.2%	1.1%	0.1%	0.748
Acute nasopharyngitis	0.6%	0.6%	0.0%	1.000
Stevens-Johnson Syndrome	*No patients observed*			

Patients were matched on age categories, sex, and GCS categories. **Bold** highlights significance at a 95% confidence level.

Conversely, gabapentin use was associated with protection against certain metabolic and cardiovascular events, including hyponatremia, hyperkalemia, and cardiac arrest. No significant differences were observed in seizures, stroke, myocardial infarction, pneumonia, or other major outcomes.

### Sensitivity analysis in moderate TBI

Using the compilation definition of moderate TBI (Supplemental Table 18), we identified 11,387 patients with moderate injuries, of which 7.3% received gabapentin. Prior to adjustment, gabapentin recipients had lower frequencies of cognitive impairment (10.0% vs. 14.1%, *P* = .001) and mortality (3.3% vs. 6.7%, *P* < .001). Multivariate modeling revealed that gabapentin was associated with a significant decrease in the hazard of mortality (HR = 0.60, 95% CI 0.41–0.88, *P* < .001; Supplemental Table 20) but no significant change in the hazard of cognitive impairment (HR = 0.82, 95% CI 0.66–1.03, *P* = .08; Supplemental Table 19).

## Discussion

Cognitive impairment is one of the most disabling long-term consequences of TBI, with no proven pharmacologic strategies for prevention. In this retrospective, real-world cohort study of 50,000 patients, we build on published methodology to demonstrate that gabapentin was associated with a significantly lower risk of durable cognitive impairment after mild TBI and with substantially reduced mortality after severe TBI. These associations, observed despite higher baseline symptom burden and comorbidities among patients prescribed gabapentin, suggest that gabapentin may exert previously unrecognized neuroprotective effects in the setting of acute brain trauma.

Should these hypothesis-generating associations be validated in prospective, randomized trials, the potential benefit from prophylactic protection against cognitive impairment and mortality cannot be overstated. Cognitive impairment is a devastating, well-studied sequelae among survivors of TBI [[1], [2], [3], [4],^48^,[56], [57], [58], [59],^63^,[66], [67], [68]]. Further, the burden of mortality after TBI is more widespread than cognitive impairment [3,4,10,14,41,[57], [58], [59],^69^,^70^]. Underlying dysfunction has been demonstrated at the ionic, molecular, synaptic, axonal, neuronal, and circuit levels [[71], [72], [73], [74]]. Inertial, mechanical injuries occur in the most vulnerable regions: the brainstem; gray-white junctions in the ventral, antero-frontal, and temporal lobes; parasagittal white matter and corpus callosum; and axons and small vasculature of the brainstem [75,76]. At smaller scales, current theories most strongly implicate glutamatergic excitotoxicity, disrupted calcium homeostasis, accumulation of cytotoxic edema, protease destruction of cytoskeletal elements, apoptosis occurring for hours to weeks after injury, and free radical accumulation [38,68,73,74,77,78]. Despite the successes in basic science, randomized trials in TBI are notoriously difficult. A systematic review with meta-analysis of randomized trials in TBI from 1978 to 2022 identified 662 such trials, of which less than half (46%) initiated a medical or surgical therapy in the acute phase after injury [48]. A similar systematic review with meta-analysis of 354 trials from 2004 to 2023 showed that 56% of trials were phase 1 or phase 2, and only 43% of trials were completed; in contrast, 38% were terminated, withdrawn, or of an indeterminable status [79].

We are not the first to suggest a role for gabapentinoids in TBI. Most recently, a trial of 90 patients randomized to standard of care, standard of care with propranolol, or standard of care with propranolol and gabapentin showed that, while propranolol reduced paroxysmal sympathetic hyperactivity, gabapentin offered additional, significant protection [37]. However, it is worth noting that the authors found longer hospital stays associated with gabapentin, which they attributed to its sedating effects. Gabapentin also was reported to reduce autonomic storming in a case series of six severe TBIs [80]. In a trial of 60 patients with severe TBI randomized to receive 300 mg gabapentin twice daily initiated in the first 24 h after injury or placebo, gabapentin was associated with decreased mortality, fewer sympathetic hyperactivity episodes, less frequent sedation boluses, lower mortality, and better Glasgow Outcome Scores at 30 and 90 days [41]. These trials help establish the groundwork for this population-level study of some 50,000 patients, and we extend the findings of these trial to include excellent generalizability, real-world use, and longer-term outcomes. Both patient-level and population-level analyses will be essential as we move toward a large-scale randomized trial of prophylactic gabapentin in TBI.

### Neurobiological underpinnings

Gabapentin has long been used for pain, agitation, and seizure prophylaxis in neurological disorders, but its potential to influence the trajectory of TBI recovery has not been established in humans. Though findings from this retrospective analysis should exclusively be interpreted as an association, a growing body of evidence suggests the possibility of a putative causal effect. Neuronal injury, such as that occurring in TBI, opens a narrow window of neuroplasticity [21]. Injury upregulates expression of thrombospondin-1, leading to excitatory synaptogenesis, but in blocking this, gabapentin appears to reduce aberrant spikes, spike bursts, and synchronized network bursts [20,22,24,81]. This effect may be mediated by removal of excitatory neurotransmitters by astrocytes in cortical layer V [82], a behavior further promoted by gabapentin [22,83,84]. Compensatory pathways begin sprouting in an attempt to compensate for deficits in the acute setting [85,86], and gabapentin encourages this diffuse plasticity [22]. Simultaneously, astrocyte- and macrophage-secreted thrombospondins bind α2δ-1 to promote excitatory synaptogenesis in autonomic pathways [29,87,88], and as gabapentin appears to block this maladaptive autonomic reinnervation, it offers protection against post-traumatic hyperalgesia, autonomic dysregulation, and dysreflexia [29,37,38,80,[89], [90], [91]]. There is also evidence that gabapentinoids may protect against posttraumatic epilepsy, implying that these medications could be re-repurposed for their original role as anti-seizure medications [19,81,[91], [92], [93], [94], [95], [96]]. These mechanisms have translated to enticing potential benefits for human patients with spinal cord injuries, particularly when gabapentinoids were administered in the minutes to hours following an injury [[32], [33], [34],^83^]. Our study provides the first population-level evidence in humans suggesting that these neuroprotective properties may translate into meaningful clinical outcomes after TBI.

### Differentiation from chronic pain literature

Recent population-based studies have linked long-term gabapentin use in the setting of managing chronic pain to an increased risk of dementia and other cognitive outcomes. These findings raise concern, but are likely confounded by the strong, bidirectional relationship between chronic pain and cognitive impairment. Patients with chronic pain show frontal cortex-mediated cognitive deficits in spite of common analgesics which are associated with decreased parvalbumin-expressing GABAergic interneurons in layers V and VI of the medial prefrontal cortex [[97], [98], [99]]. While gabapentin repairs the deficit of parvalbumin-expressing interneurons, this effect alone may not be sufficient to ameliorate the cognitive deficits associated with chronic pain [100]. The changes associated with chronic pain and chronic gabapentin use critically differ from the pathophysiology at play in acute neurotrauma, and therefore may not detract from neuroprotective effects of gabapentin in the acute setting following TBI. This being said, we observed significant differences in long-term outcomes between gabapentin recipients and non-recipients (e.g., reduced mobility, sleep disorders, mental health conditions, cardiovascular complications), suggesting a more complex interplay of factors that goes beyond “protective vs. harmful” and necessitates further study.

### Interpretations

Levetiracetam was prescribed to a third of patients irrespective of whether they received gabapentin, and while no association with cognitive impairment was observed, levetiracetam and gabapentin were both associated with lower mortality risks in severe TBI. Levetiracetam recipients may have received an overall higher level of care than non-recipients. Levetiracetam is recommended as early seizure prophylaxis in severe TBI [[7], [8], [9]], but no differences in long-term outcomes have been reported [9,[11], [12], [13]]. That a long-term protective association between levetiracetam and mortality was found here, but not in many prior high-quality studies, suggests that overall quality of care may have been higher in levetiracetam recipients as opposed to a primary drug effect. This explanation may be extrapolated to the gabapentin observations as well. As we could not stratify by prescriber or healthcare organization, it is possible that gabapentin was prescribed by more proactive, attentive, or aggressive physicians. Gabapentin was likely administered to the study population for neuropathic pain or agitation [[15], [16], [17], [18],^81^], and may have been co-administered with other treatments unaccounted for here that contributed to the better prognosis observed.

Though an overall association between gabapentin and decrease cognitive impairment was observed in mild TBI, the association was significant at the 100 mg dose but not at the 300 mg dose. The reasons for this remain unclear but may be attributable to various confounding effects. Patients receiving the 300 mg dose may have had more severe injuries than patients receiving the 100 mg dose. Similarly, the 300 mg dose may have been prescribed in response to more severe symptoms than the 100 mg dose. Alternatively, the 100 mg dose may have been just sufficient to promote the development of compensatory pathways and suppress maladaptive neuroexcitability [22,85,86], while the 300 mg dose may have been enough to activate microglia at the α2δ-1 receptor, thereby exacerbating neuroinflammation and inhibiting neuroplasticity to a detrimental degree [16,20].

### Limitations

Several important limitations merit consideration. First, this was a retrospective analysis of electronic health record data, and residual confounding (e.g., confounding by indication) is inevitable despite adjustment for multiple clinical variables and comorbidities, especially as patients who were prescribed gabapentin differed in many aspects from those who were not. Second, durable cognitive impairment was defined by an amalgam of ICD-10 codes rather than standardized neuropsychological testing, raising the possibility of misclassification errors [101]. Third, gabapentin prescription details such as indication, frequency, cumulative dose, and treatment duration were not available; as previously published, these factors may have critically influenced outcomes [16]. Fourth, patients prescribed gabapentin differed systematically at baseline from those who were not, including a greater prevalence of pain, psychiatric symptoms, and polypharmacy, potentially influencing outcomes in unknowable and complex ways. Fifth, GCS scores were recorded in only a subset of patients in the TriNetX network, which may limit generalizability and introduce selection bias. Likewise, 2.6% of patients were counted in multiple GCS categories, though it is likely that this would bias results toward the null. Sixth, moderate TBI could not be defined purely by GCS scores, and while our compiled moderate definition showed intermediate findings between mild and severe TBI, this remains an area of ambiguity. Seventh, we were unable to account for mechanism of injury [13,69]. Eighth, we assessed comorbidities using diagnoses present in the electronic medical records, and as obtunded patients may have been unable to provide updates or verify information in the records, which may have led to a potential differential information bias. Lastly, as with all observational studies, causality cannot be inferred; despite their statistical and clinical significance, our findings should be interpreted as hypothesis-generating and requiring prospective confirmation. Our observed association alone does not support changes to the standard of care for TBI, and gabapentin should not be initiated solely for neuroprotection outside of an approved clinical trial.

### Clinical implications

Despite these limitations, we present a large, robust analysis with strong external validity that demonstrates a biologically plausible association between gabapentin and decreased cognitive impairment after TBI. If validated prospectively, these findings could represent an important shift in the acute management of TBI. Treatment of disordered cognition and consciousness currently emphasizes symptom-based care because no pharmacologic agent has demonstrated the potential for prophylactic reduction of the risk of long-lasting cognitive decline [6]. Gabapentin is inexpensive, widely available, and well tolerated, with more than three decades of use in neurology and beyond. Its association with reduced cognitive impairment in mild TBI suggests that gabapentin could fill a critical therapeutic gap for post-traumatic patients at risk of long-term disability. Furthermore, the nearly 50% reduction in mortality among patients with severe TBI underscores gabapentin’s potential relevance in neurocritical care, where therapeutic options are limited and outcomes are often dismal.

### Conclusions

Gabapentin, long relegated to symptomatic use in chronic pain, may hold untapped potential as the first widely accessible pharmacologic strategy to reduce the burden of cognitive decline and death after TBI. In this large, real-world cohort, gabapentin was associated with a lower risk of durable cognitive impairment after mild TBI and nearly half the risk of mortality after severe TBI. While these findings cannot establish causality, they compel a re-examination of gabapentin not merely as a pain or seizure prevention drug, but as a candidate neuroprotective therapy. Prospective, randomized trials are now urgently warranted to determine whether this familiar agent can translate its observed associations into meaningful advances in the treatment of TBI.

## Statements and Declarations

The authors have nothing to report.

## Data availability

The data that support the findings of this study are available from TriNetX, LLC but third-party restrictions apply to the availability of these data. The data were used under license for this study with restrictions that do not allow for the data to be redistributed or made publicly available. However, for accredited researchers, the TriNetX data is available for licensing at TriNetX, LLC. Data access may require a data sharing agreement and may incur data access fees.

## Author contributions

IBT, AS, AM, EF, PEM, MCS, DJK, SMW, AB, SI, SAM, CDK, and FAM contributed to the conception and design of the study. IBT, AS, AM, JAM, CRM, ME, TRG, MDK, JBR, AB, SI, SAM, CDK, and FAM contributed to the acquisition and analysis of data. IBT, AS, AG, AGL, AM, EF, JAM, PEM, MCS, CRM, DJK, ME, SMW, TRG, MDK, JBR, AB, SI, SAM, CDG, and FAM contributed to drafting the text or preparing the figures.

## Declaration of competing interest

The authors declare that they have no known competing financial interests or personal relationships that could have appeared to influence the work reported in this paper.
