# Supplementary material for: Gabapentin and cognitive impairment after traumatic brain injury: A multinational cohort of 49,925 patients
Source: Neurotherapeutics. 2026 Aug 13;23(5):e01041. doi: 10.1016/j.neurot.2026.e01041 (PMC13615720; doi:10.1016/j.neurot.2026.e01041)
Supplement: Supplementary file 1 [file mmc1.docx]

# Supplemental Table 1. Medical History Up To and Including Day of TBI

* Cerebrovascular conditions were assessed up to the day prior to TBI, but not on the day of TBI. **Bold** indicates significance at 95% confidence level.

| **Characteristic Name** | **Gabapentin** | | **No Gabapentin** | | **p-value** |
| --- | --- | --- | --- | --- | --- |
|  | **Patients** | **Frequency** | **Patients** | **Frequency** |  |
| Chronic viral hepatitis | 21 | 1.2% | 508 | 1.1% | 0.9001 |
| Human immunodeficiency virus [HIV] disease | 11 | 0.6% | 193 | 0.4% | 0.0496 |
| Malignancies of lip, oral cavity and pharynx | 1-10 | 0.6% | 86 | 0.2% |  |
| Malignancies of digestive organs | 1-10 | 0.6% | 355 | 0.7% |  |
| Malignancies of respiratory and intrathoracic organs | 1-10 | 0.6% | 231 | 0.5% |  |
| Malignancies of bone and articular cartilage | 0 | 0.0% | 24 | 0.1% | 0.4177 |
| Melanoma and other malignancies of skin | 18 | 1.0% | 723 | 1.5% | 0.1468 |
| Malignancies of mesothelial and soft tissue | 1-10 | 0.6% | 60 | 0.1% |  |
| Malignant neoplasm of breast | 1-10 | 0.6% | 299 | 0.6% |  |
| Malignancies of female genital organs | 1-10 | 0.6% | 79 | 0.2% |  |
| Malignancies of male genital organs | 13 | 0.7% | 418 | 0.9% | 0.5928 |
| Malignancies of urinary tract | 1-10 | 0.6% | 204 | 0.4% |  |
| Malignancies of eye, brain and other parts of central nervous system | 1-10 | 0.6% | 116 | 0.2% |  |
| Malignancies of thyroid and other endocrine glands | 1-10 | 0.6% | 66 | 0.1% |  |
| Malignancies of ill-defined, other secondary and unspecified sites | 12 | 0.7% | 601 | 1.3% | 0.1825 |
| Malignancies of lymphoid, hematopoietic and related tissue | 13 | 0.7% | 451 | 0.9% | 0.8032 |
| Diabetes mellitus | 282 | 16.1% | 6,206 | 12.9% | 0.1934 |
| **Delirium due to known physiological condition** | **51** | **2.9%** | **1,240** | **2.6%** | **0.0031** |
| Transient cerebral ischemic attacks and related syndromes | 20 | 1.1% | 571 | 1.2% | 0.5075 |
| Vascular syndromes of brain in cerebrovascular diseases | 1-10 | 0.6% | 42 | 0.1% |  |
| Cerebral palsy and other paralytic syndromes | 89 | 5.1% | 2,400 | 5.0% | 0.3205 |
| Acute myocardial infarction | 47 | 2.7% | 1,422 | 3.0% | 0.3082 |
| Subsequent ST elevation (STEMI) and non-ST elevation (NSTEMI) myocardial infarction | 0 | 0.0% | 15 | 0.0% | 0.5082 |
| Chronic ischemic heart disease | 209 | 11.9% | 5,036 | 10.5% | 0.0560 |
| Other pulmonary heart diseases | 34 | 1.9% | 1,051 | 2.2% | 0.3783 |
| **Cardiomyopathy** | **26** | **1.5%** | **1,006** | **2.1%** | **0.0344** |
| Cardiomyopathy in diseases classified elsewhere | 1-10 | 0.6% | 49 | 0.1% |  |
| Heart failure | 112 | 6.4% | 2,742 | 5.7% | 0.0789 |
| **Anxiety disorders** | **363** | **20.7%** | **8,151** | **16.9%** | **<0.0001** |
| **Depressive episode** | **277** | **15.8%** | **548** | **13.1%** | **0.0020** |
| Schizophrenia spectrum disorders | 35 | 2.0% | 1,248 | 2.6% | 0.1375 |
| Major depressive disorder, recurrent | 33 | 1.9% | 996 | 2.1% | 0.6001 |
| *Nontraumatic subarachnoid hemorrhage | 1-10 | 0.6% | 224 | 0.5% |  |
| *Nontraumatic intracerebral hemorrhage | 1-10 | 0.6% | 274 | 0.6% |  |
| *Other and unspecified nontraumatic intracranial hemorrhage | 1-10 | 0.6% | 259 | 0.5% |  |
| *Cerebral infarction | 26 | 1.5% | 845 | 1.8% | 0.3886 |
| *Occlusion and stenosis of precerebral arteries, not resulting in cerebral infarction | 18 | 1.0% | 700 | 1.5% | 0.1383 |
| *Occlusion and stenosis of cerebral arteries, not resulting in cerebral infarction | 1-10 | 0.6% | 62 | 0.1% |  |
| *Other cerebrovascular diseases | 16 | 0.9% | 664 | 1.4% | 0.0967 |
| *Cerebrovascular disorders in diseases classified elsewhere | 1-10 | 0.6% | 12 | 0.0% |  |
| *Sequelae of cerebrovascular disease | 11 | 0.6% | 408 | 0.8% | 0.3189 |
| **Atherosclerosis** | **45** | **2.6%** | **1,496** | **3.1%** | **0.0076** |
| Aortic aneurysm and dissection | 38 | 2.2% | 901 | 1.9% | 0.3289 |
| Other peripheral vascular diseases | 45 | 2.6% | 1,055 | 2.2% | 0.1688 |
| Disorders of arteries, arterioles and capillaries in diseases classified elsewhere | 0 | 0.0% | 26 | 0.1% | 0.3395 |
| Esophageal varices | 1-10 | 0.6% | 222 | 0.5% |  |
| **Chronic lower respiratory diseases** | **246** | **14.0%** | **6,394** | **13.3%** | **0.0273** |
| Lung diseases due to external agents | 44 | 2.5% | 2,224 | 4.6% | 0.9928 |
| Gastric ulcer | 1-10 | 0.6% | 334 | 0.7% |  |
| Duodenal ulcer | 1-10 | 0.6% | 194 | 0.4% |  |
| Peptic ulcer, site unspecified | 1-10 | 0.6% | 181 | 0.4% |  |
| Gastrojejunal ulcer | 0 | 0.0% | 25 | 0.1% | 0.3814 |
| Vascular disorders of intestine | 1-10 | 0.6% | 267 | 0.6% |  |
| Alcoholic liver disease | 26 | 1.5% | 693 | 1.4% | 0.7729 |
| Toxic liver disease | 1-10 | 0.6% | 70 | 0.1% |  |
| Hepatic failure, not elsewhere classified | 1-10 | 0.6% | 550 | 1.1% |  |
| Chronic hepatitis, not elsewhere classified | 1-10 | 0.6% | 46 | 0.1% |  |
| Fibrosis and cirrhosis of liver | 28 | 1.6% | 718 | 1.5% | 0.8939 |
| Other diseases of liver | 105 | 6.0% | 2,701 | 5.6% | 0.3103 |
| Rheumatoid arthritis with rheumatoid factor | 1-10 | 0.6% | 86 | 0.2% |  |
| Other rheumatoid arthritis | 23 | 1.3% | 443 | 0.9% | 0.9982 |
| Systemic connective tissue disorders | 15 | 0.9% | 390 | 0.8% | 0.9392 |
| Chronic nephritic syndrome | 0 | 0.0% | 20 | 0.0% | 0.3930 |
| Unspecified nephritic syndrome | 0 | 0.0% | 58 | 0.1% | 0.1565 |
| Chronic kidney disease (CKD) | 94 | 5.4% | 2,746 | 5.7% | 0.0599 |
| Unspecified kidney failure | 1-10 | 0.6% | 221 | 0.5% |  |
| Encounter for care involving renal dialysis | 1-10 | 0.6% | 42 | 0.1% |  |
| Transplanted organ and tissue status | 1-10 | 0.6% | 234 | 0.5% |  |
| **Presence of cardiac and vascular implants and grafts** | **129** | **7.4%** | **3,188** | **6.6%** | **0.0079** |

# Supplemental Table 2. Characteristics on the Day of TBI

Characteristics were obtained on the day of TBI.

| **Characteristic** | **Gabapentin** | | **No Gabapentin** | | **p-value** |
| --- | --- | --- | --- | --- | --- |
|  | **Patients** | **Frequency** | **Patients** | **Frequency** |  |
| *Demographics* | | | | | |
| Age at Index (mean ± standard deviation years old) | 1,733 | 51.6 ± 18.8 | 47,287 | 48.4 ± 20.3 | **<0.0001** |
| Male | 1,061 | 61.2% | 30,467 | 64.4% | **0.0062** |
| Female | 525 | 30.3% | 14,633 | 30.9% | 0.5648 |
| *Neurologic and psychiatric symptoms and diagnoses* | | | | | |
| Mental and behavioral disorders due to psychoactive substance use | 721 | 41.6% | 16,652 | 35.2% | **<0.0001** |
| Mood [affective] disorders | 288 | 16.6% | 5,068 | 10.7% | **<0.0001** |
| Anxiety, dissociative, stress-related, somatoform and other nonpsychotic mental disorders | 290 | 16.7% | 4,899 | 10.4% | **<0.0001** |
| Pain, not elsewhere classified | 670 | 38.7% | 7,612 | 16.1% | **<0.0001** |
| Acute pain due to trauma | 578 | 33.4% | 6,113 | 12.9% | **<0.0001** |
| Nontraumatic subarachnoid hemorrhage | 317 | 18.3% | 8,081 | 17.1% | 0.1919 |
| Nontraumatic intracerebral hemorrhage | 89 | 5.1% | 3,869 | 8.2% | **<0.0001** |
| Other and unspecified nontraumatic intracranial hemorrhage | 285 | 16.4% | 7,829 | 16.6% | 0.9029 |
| Cerebral infarction | 51 | 2.9% | 1,629 | 3.4% | 0.2592 |
| Occlusion and stenosis of precerebral arteries, not resulting in cerebral infarction | 58 | 3.3% | 1,331 | 2.8% | 0.1898 |
| Occlusion and stenosis of cerebral arteries, not resulting in cerebral infarction | 1-10 | 0.6% | 165 | 0.3% |  |
| Other cerebrovascular diseases | 42 | 2.4% | 1,331 | 2.8% | 0.3324 |
| Cerebrovascular disorders in diseases classified elsewhere | 0 | 0.0% | 62 | 0.1% | 0.1315 |
| Sequelae of cerebrovascular disease | 31 | 1.8% | 707 | 1.5% | 0.3241 |
| Abnormalities of heartbeat | 249 | 14.4% | 7,179 | 15.2% | 0.3535 |
| Abdominal and pelvic pain | 63 | 3.6% | 1,895 | 4.0% | 0.4372 |
| Nausea and vomiting | 54 | 3.1% | 1,930 | 4.1% | **0.0452** |
| Aphagia and dysphagia | 170 | 9.8% | 4,536 | 9.6% | 0.7632 |
| Disturbances of skin sensation | 70 | 4.0% | 655 | 1.4% | **<0.0001** |
| Abnormalities of gait and mobility | 83 | 4.8% | 1,330 | 2.8% | **<0.0001** |
| Retention of urine | 191 | 11.0% | 2,745 | 5.8% | **<0.0001** |
| Somnolence | 78 | 4.5% | 687 | 1.5% | **<0.0001** |
| Stupor | 70 | 4.0% | 522 | 1.1% | **<0.0001** |
| Other symptoms and signs involving cognitive functions and awareness | 350 | 20.2% | 7,872 | 16.6% | **0.0001** |
| Disorientation, unspecified | 55 | 3.2% | 1,885 | 4.0% | 0.0883 |
| Altered mental status, unspecified | 102 | 5.9% | 3,846 | 8.1% | **0.0007** |
| Cognitive communication deficit | 111 | 6.4% | 938 | 2.0% | **<0.0001** |
| Headache | 82 | 4.7% | 2,528 | 5.3% | 0.2632 |
| Pain, unspecified | 61 | 3.5% | 1,205 | 2.5% | **0.0123** |
| Malaise and fatigue | 78 | 4.5% | 1,614 | 3.4% | **0.0148** |
| Syncope and collapse | 113 | 6.5% | 2,836 | 6.0% | 0.3684 |
| Elevated blood glucose level | 141 | 8.1% | 4,080 | 8.6% | 0.4733 |
| *Types of non-neurologic injuries* | | | | | |
| Superficial injury of head | 385 | 22.2% | 10,206 | 21.6% | 0.5296 |
| Superficial injury of scalp | 174 | 10.0% | 4,587 | 9.7% | 0.6387 |
| Open wound of head | 615 | 35.5% | 15,766 | 33.3% | 0.0628 |
| Open wound of scalp | 317 | 18.3% | 7,529 | 15.9% | **0.0082** |
| Open wound of eyelid and periocular area | 136 | 7.8% | 2,960 | 6.3% | **0.0076** |
| Open wound of ear | 50 | 2.9% | 1,069 | 2.3% | 0.0873 |
| Open wound of cheek and temporomandibular area | 35 | 2.0% | 679 | 1.4% | **0.0464** |
| Open wound of lip and oral cavity | 91 | 5.3% | 2,047 | 4.3% | 0.0649 |
| Fracture of skull and facial bones | 541 | 31.2% | 16,331 | 34.5% | **0.0043** |
| Fracture of vault of skull | 129 | 7.4% | 4,591 | 9.7% | **0.0017** |
| Fracture of base of skull | 313 | 18.1% | 9,661 | 20.4% | 0.0161 |
| Fracture of nasal bones | 177 | 10.2% | 4,603 | 9.7% | 0.5088 |
| Fracture of malar, maxillary and zygoma bones | 214 | 12.3% | 5,782 | 12.2% | 0.8799 |
| Fracture of mandible | 62 | 3.6% | 1,610 | 3.4% | 0.6970 |
| Injury of eye and orbit | 74 | 4.3% | 2,297 | 4.9% | 0.2629 |
| Injuries to the neck | 520 | 30.0% | 10,879 | 23.0% | **<0.0001** |
| Injuries to the abdomen, lower back, lumbar spine, pelvis and external genitals | 747 | 43.1% | 14,655 | 31.0% | **<0.0001** |
| Injuries to the shoulder and upper arm | 523 | 30.2% | 8,724 | 18.4% | **<0.0001** |
| Injuries to the elbow and forearm | 307 | 17.7% | 6,146 | 13.0% | **<0.0001** |
| Injuries to the wrist, hand and fingers | 301 | 17.4% | 5,772 | 12.2% | **<0.0001** |
| Injuries to the hip and thigh | 249 | 14.4% | 4,670 | 9.9% | **<0.0001** |
| Injuries to the ankle and foot | 192 | 11.1% | 2,961 | 6.3% | **<0.0001** |
| Multiple sites of injury | 185 | 10.7% | 3,204 | 6.8% | **<0.0001** |
| *Mechanism of injury (if recorded)* | | | | | |
| Motorcycle rider injured in transport accident | 154 | 8.9% | 3,085 | 6.5% | **0.0001** |
| Car occupant injured in transport accident | 353 | 20.4% | 7,410 | 15.7% | **<0.0001** |
| Slipping, tripping, stumbling and falls | 501 | 28.9% | 13,852 | 29.3% | 0.7300 |
| Assault | 79 | 4.6% | 3,079 | 6.5% | **0.0011** |
| Street, highway and other paved roadways as the place of occurrence of the external cause | 482 | 27.8% | 9,181 | 19.4% | **<0.0001** |
| *Laboratory and diagnostic testing performed* | | | | | |
| Computed tomography, head or brain | 1,143 | 66.0% | 33,458 | 70.8% | **<0.0001** |
| Hematology and coagulation procedures | 1,095 | 63.2% | 30,379 | 64.2% | 0.3666 |
| Organ or disease oriented panels | 1,023 | 59.0% | 28,310 | 59.9% | 0.4847 |
| Chemistry procedures | 954 | 55.0% | 27,110 | 57.3% | 0.0593 |
| Electrocardiogram, routine ECG with at least 12 leads | 588 | 33.9% | 15,072 | 31.9% | 0.0714 |
| Diagnostic ultrasound procedures of the abdomen and retroperitoneum | 408 | 23.5% | 7,659 | 16.2% | **<0.0001** |
| Echocardiography procedures | 352 | 20.3% | 7,102 | 15.0% | **<0.0001** |
| Diagnostic ultrasound procedures of the chest | 315 | 18.2% | 4,833 | 10.2% | **<0.0001** |
| Computed tomographic angiography, head, with contrast material(s), including noncontrast images, if performed, and image postprocessing | 254 | 14.7% | 6,638 | 14.0% | 0.4666 |
| Surgical procedures on the musculoskeletal system | 221 | 12.8% | 2,658 | 5.6% | **<0.0001** |
| *Medical management* | | | | | |
| Analgesics | 1,688 | 97.4% | 34,455 | 72.9% | **<0.0001** |
| Opioid analgesics | 1,570 | 90.6% | 28,194 | 59.6% | **<0.0001** |
| Therapeutic nutrients/minerals/electrolytes | 1,549 | 89.4% | 31,430 | 66.5% | **<0.0001** |
| Acetaminophen | 1,520 | 87.7% | 21,345 | 45.1% | **<0.0001** |
| Gastrointestinal medications | 1,512 | 87.2% | 28,045 | 59.3% | **<0.0001** |
| Respiratory tract medications | 1,500 | 86.6% | 31,116 | 65.8% | **<0.0001** |
| Pharmaceutical aids/reagents | 1,478 | 85.3% | 30,103 | 63.7% | **<0.0001** |
| Irrigation/dialysis solutions | 1,460 | 84.2% | 29,799 | 63.0% | **<0.0001** |
| Laxatives, rectal | 829 | 47.8% | 12,155 | 25.7% | **<0.0001** |
| Antiarrhythmics | 778 | 44.9% | 13,204 | 27.9% | **<0.0001** |
| Lidocaine | 770 | 44.4% | 12,908 | 27.3% | **<0.0001** |
| Methocarbamol | 610 | 35.2% | 1,779 | 3.8% | **<0.0001** |
| Antihypoglycemics | 586 | 33.8% | 8,643 | 18.3% | **<0.0001** |
| Levetiracetam | 564 | 32.5% | 15,390 | 32.5% | 0.9991 |
| Benzodiazepine derivative sedatives/hypnotics | 550 | 31.7% | 10,863 | 23.0% | **<0.0001** |
| Anticoagulants | 547 | 31.6% | 5,075 | 10.7% | **<0.0001** |
| Beta blockers/related | 461 | 26.6% | 7,241 | 15.3% | **<0.0001** |
| Naloxone | 359 | 20.7% | 3,120 | 6.6% | **<0.0001** |
| Propofol | 343 | 19.8% | 11,073 | 23.4% | **0.0005** |
| Enoxaparin | 365 | 21.1% | 2,273 | 4.8% | **<0.0001** |
| Glucocorticoids | 336 | 19.4% | 6,124 | 13.0% | **<0.0001** |
| Insulin | 307 | 17.7% | 4,788 | 10.1% | **<0.0001** |
| Antidepressants | 275 | 15.9% | 2,045 | 4.3% | **<0.0001** |
| Rocuronium | 222 | 12.8% | 5,935 | 12.6% | 0.7492 |
| Ibuprofen | 195 | 11.3% | 2,143 | 4.5% | **<0.0001** |
| Bupivacaine | 173 | 10.0% | 3,935 | 8.3% | **0.0142** |
| Platelet aggregation inhibitors | 176 | 10.2% | 1,622 | 3.4% | **<0.0001** |
| Heparin | 168 | 9.7% | 2,485 | 5.3% | **<0.0001** |
| Aspirin | 160 | 9.2% | 1,505 | 3.2% | **<0.0001** |
| Cyclobenzaprine | 157 | 9.1% | 1,000 | 2.1% | **<0.0001** |
| Ketorolac | 134 | 7.7% | 1,558 | 3.3% | **<0.0001** |
| Antipsychotics | 132 | 7.6% | 2,210 | 4.7% | **<0.0001** |
| Baclofen | 27 | 1.6% | 62 | 0.1% | **<0.0001** |
| Orphenadrine | 22 | 1.3% | 111 | 0.2% | **<0.0001** |
| Tizanidine | 21 | 1.2% | 75 | 0.2% | **<0.0001** |

# Supplemental Table 3. All GCS, Risk of Durable Cognitive Impairment by 2 Years

| **Covariate** | **Hazard Ratio** | **95 % CI Lower** | **95 % CI Upper** | **P > \|z\|** |
| --- | --- | --- | --- | --- |
| Gabapentin (vs. no gabapentin) | 0.8783 | 0.7258 | 1.0630 | 0.1827 |
| GCS 13-15 | 0.9537 | 0.8097 | 1.1233 | 0.5701 |
| **GCS 9-12** | **1.4148** | **1.2146** | **1.6480** | **<0.0001** |
| GCS 3-8 | 1.1314 | 0.9571 | 1.3375 | 0.1482 |
| Age at Index [REF = patients 18-30 years old] |  |  |  |  |
| 30 - 35 Years | 1.0646 | 0.8485 | 1.3357 | 0.5888 |
| 35 - 40 Years | 0.9743 | 0.7564 | 1.2550 | 0.8401 |
| 40 - 45 Years | 1.2664 | 0.9995 | 1.6046 | 0.0505 |
| **45 - 50 Years** | **1.5692** | **1.2627** | **1.9500** | **<0.0001** |
| **50 - 55 Years** | **1.5253** | **1.2365** | **1.8815** | **0.0001** |
| **55 - 60 Years** | **1.9908** | **1.6503** | **2.4017** | **<0.0001** |
| **60 - 65 Years** | **2.5689** | **2.1593** | **3.0563** | **<0.0001** |
| **65 - 70 Years** | **3.4464** | **2.9231** | **4.0634** | **<0.0001** |
| **70 - 75 Years** | **5.3433** | **4.5747** | **6.2410** | **<0.0001** |
| **75 - 80 Years** | **7.5165** | **6.4595** | **8.7463** | **<0.0001** |
| **≥80 Years** | **12.7835** | **11.0717** | **14.7599** | **<0.0001** |
| Male (vs. Female) | 1.0062 | 0.9386 | 1.0786 | 0.8626 |
| **Concussion** | **0.6246** | **0.5503** | **0.7090** | **<0.0001** |
| Traumatic cerebral edema | 1.0985 | 0.9821 | 1.2287 | 0.1004 |
| **Diffuse traumatic brain injury** | **1.3264** | **1.2027** | **1.4629** | **<0.0001** |
| Epidural hemorrhage | 1.0603 | 0.9157 | 1.2276 | 0.4339 |
| **Traumatic subdural hemorrhage** | **1.2511** | **1.1555** | **1.3545** | **<0.0001** |
| **Traumatic subarachnoid hemorrhage** | **1.1072** | **1.0185** | **1.2037** | **0.0169** |
| **Traumatic brain compression and herniation** | **0.7754** | **0.6601** | **0.9108** | **0.0019** |
| Unspecified focal traumatic brain injury | 0.9583 | 0.8251 | 1.1132 | 0.5776 |
| Contusion and laceration of right cerebrum | 0.9542 | 0.7706 | 1.1816 | 0.6674 |
| **Contusion and laceration of left cerebrum** | **1.2800** | **1.0489** | **1.5621** | **0.0151** |
| **Traumatic hemorrhage of right cerebrum** | **1.2321** | **1.0588** | **1.4338** | **0.0070** |
| Traumatic hemorrhage of left cerebrum | 0.9173 | 0.7766 | 1.0834 | 0.3094 |
| Contusion, laceration, or hemorrhage of brainstem | 0.9502 | 0.6489 | 1.3913 | 0.7928 |
| Fracture of skull and facial bones | 0.9755 | 0.9002 | 1.0572 | 0.5457 |
| Dislocation/sprain of joints/ligaments at neck level | 1.1652 | 0.9364 | 1.4499 | 0.1704 |
| **Injury of nerves and spinal cord at neck level** | **0.6690** | **0.5217** | **0.8580** | **0.0015** |
| Blood Pressure, Systolic (<90 mmHg vs. ≥90 mmHg) | 1.0042 | 0.8908 | 1.1320 | 0.9459 |
| Nontraumatic subarachnoid hemorrhage | 1.0000 | 0.9136 | 1.0946 | 0.9996 |
| Nontraumatic intracerebral hemorrhage | 1.0631 | 0.9575 | 1.1803 | 0.2517 |
| Unspecified nontraumatic intracranial hemorrhage | 1.0206 | 0.9405 | 1.1076 | 0.6250 |
| **Cerebral infarction** | **1.1670** | **1.0190** | **1.3364** | **0.0256** |
| Physiological Systems / Performance / Respiratory | 1.0263 | 0.9229 | 1.1414 | 0.6314 |
| Craniectomy or Craniotomy Procedures | 0.9822 | 0.8250 | 1.1694 | 0.8399 |
| Twist Drill, Burr Hole(s), or Trephine Procedures | 1.1632 | 0.9360 | 1.4456 | 0.1728 |
| **Routine Electroencephalography (EEG) Procedures** | **1.6584** | **1.3352** | **2.0598** | **<0.0001** |
| **Sympathomimetics** | **0.8789** | **0.7985** | **0.9674** | **0.0084** |
| Transfusion, blood or blood components | 0.9584 | 0.8291 | 1.1079 | 0.5655 |
| Emergency Department Services | 0.9406 | 0.8787 | 1.0068 | 0.0777 |
| **Disorientation, amnesia** | **1.5880** | **1.4692** | **1.7164** | **<0.0001** |
| Stupor | 0.9284 | 0.6943 | 1.2413 | 0.6160 |
| Malaise and fatigue | 1.0048 | 0.8758 | 1.1527 | 0.9456 |
| **Abnormal movements, gait disturbance** | **1.6364** | **1.5016** | **1.7833** | **<0.0001** |
| Alcohol related disorders | 1.1367 | 1.0350 | 1.2484 | 0.0074 |

# Supplemental Table 4. GCS 13-15, Risk of Durable Cognitive Impairment by 2 Years

| **Covariate** | **Hazard Ratio** | **95 % CI Lower** | **95 % CI Upper** | **P > \|z\|** |
| --- | --- | --- | --- | --- |
| **Gabapentin (vs. no gabapentin)** | **0.7801** | **0.6235** | **0.9760** | **0.0298** |
| Age at Index [REF = patients 18-30 years old] |  |  |  |  |
| 30 - 35 Years | 1.4938 | 0.9681 | 2.3050 | 0.0698 |
| **35 - 40 Years** | **1.8780** | **1.2327** | **2.8612** | **0.0033** |
| **40 - 45 Years** | **2.1223** | **1.3983** | **3.2212** | **0.0004** |
| **45 - 50 Years** | **2.6281** | **1.7798** | **3.8809** | **<0.0001** |
| **50 - 55 Years** | **3.2136** | **2.2596** | **4.5704** | **<0.0001** |
| **55 - 60 Years** | **4.3169** | **3.1275** | **5.9585** | **<0.0001** |
| **60 - 65 Years** | **6.3377** | **4.6843** | **8.5747** | **<0.0001** |
| **65 - 70 Years** | **9.9448** | **7.4644** | **13.2493** | **<0.0001** |
| **70 - 75 Years** | **13.8373** | **10.4529** | **18.3174** | **<0.0001** |
| **75 - 80 Years** | **19.5168** | **14.8014** | **25.7343** | **<0.0001** |
| **≥80 Years** | **34.5141** | **26.3477** | **45.2117** | **<0.0001** |
| Male (vs. Female) | 1.0354 | 0.9505 | 1.1278 | 0.4256 |
| **Concussion** | **0.6637** | **0.5728** | **0.7690** | **<0.0001** |
| **Traumatic cerebral edema** | **1.2219** | **1.0395** | **1.4365** | **0.0151** |
| **Diffuse traumatic brain injury** | **1.1671** | **1.0091** | **1.3499** | **0.0373** |
| Epidural hemorrhage | 1.0778 | 0.8776 | 1.3238 | 0.4747 |
| **Traumatic subdural hemorrhage** | **1.1640** | **1.0540** | **1.2855** | **0.0027** |
| Traumatic subarachnoid hemorrhage | 1.0990 | 0.9865 | 1.2243 | 0.0867 |
| Traumatic brain compression and herniation | 0.9732 | 0.7827 | 1.2101 | 0.8068 |
| Unspecified focal traumatic brain injury | 1.0918 | 0.8966 | 1.3295 | 0.3820 |
| Contusion and laceration of right cerebrum | 0.9449 | 0.7075 | 1.2620 | 0.7012 |
| Contusion and laceration of left cerebrum | 1.2655 | 0.9568 | 1.6739 | 0.0989 |
| **Traumatic hemorrhage of right cerebrum** | **1.2238** | **1.0020** | **1.4948** | **0.0478** |
| Traumatic hemorrhage of left cerebrum | 1.1059 | 0.8912 | 1.3723 | 0.3605 |
| Contusion, laceration, or hemorrhage of brainstem | 1.0168 | 0.5737 | 1.8022 | 0.9546 |
| Fracture of skull and facial bones | 0.9355 | 0.8435 | 1.0375 | 0.2066 |
| Dislocation/sprain of joints/ligaments at neck level | 1.0601 | 0.7665 | 1.4661 | 0.7244 |
| Injury of nerves and spinal cord at neck level | 0.7211 | 0.5107 | 1.0180 | 0.0631 |
| Blood Pressure, Systolic (<90 mmHg vs. ≥90 mmHg) | 1.0550 | 0.8697 | 1.2797 | 0.5869 |
| Nontraumatic subarachnoid hemorrhage | 1.0290 | 0.9143 | 1.1580 | 0.6356 |
| **Nontraumatic intracerebral hemorrhage** | **1.1838** | **1.0321** | **1.3577** | **0.0158** |
| Unspecified nontraumatic intracranial hemorrhage | 1.0746 | 0.9697 | 1.1910 | 0.1700 |
| **Cerebral infarction** | **1.2556** | **1.0451** | **1.5086** | **0.0151** |
| Physiological Systems / Performance / Respiratory | 1.1284 | 0.9353 | 1.3614 | 0.2072 |
| Craniectomy or Craniotomy Procedures | 1.1014 | 0.8159 | 1.4867 | 0.5282 |
| Twist Drill, Burr Hole(s), or Trephine Procedures | 1.0037 | 0.6516 | 1.5459 | 0.9868 |
| **Routine Electroencephalography (EEG) Procedures** | **1.7451** | **1.2334** | **2.4690** | **0.0017** |
| Sympathomimetics | 0.9062 | 0.7950 | 1.0331 | 0.1408 |
| Transfusion, blood or blood components | 0.8748 | 0.6893 | 1.1103 | 0.2714 |
| Emergency Department Services | 0.9477 | 0.8711 | 1.0310 | 0.2116 |
| **Disorientation, amnesia** | **1.7351** | **1.5701** | **1.9176** | **<0.0001** |
| Stupor | 0.9217 | 0.6091 | 1.3949 | 0.6999 |
| Malaise and fatigue | 0.9372 | 0.7896 | 1.1124 | 0.4580 |
| **Abnormal movements, gait disturbance** | **1.5713** | **1.4146** | **1.7453** | **<0.0001** |
| **Alcohol related disorders** | **1.1425** | **1.0075** | **1.2956** | **0.0378** |

# Supplemental Table 5. GCS 3-8, Risk of Durable Cognitive Impairment by 2 Years

| **Covariate** | **Hazard Ratio** | **95 % CI Lower** | **95 % CI Upper** | **P > \|z\|** |
| --- | --- | --- | --- | --- |
| Gabapentin (vs. no gabapentin) | 1.1328 | 0.6992 | 1.8355 | 0.6125 |
| Age at Index [REF = patients 18-30 years old] |  |  |  |  |
| 30 - 35 Years | 1.0574 | 0.7971 | 1.4026 | 0.6988 |
| 35 - 40 Years | 0.7907 | 0.5546 | 1.1272 | 0.1943 |
| 40 - 45 Years | 1.0953 | 0.7917 | 1.5152 | 0.5825 |
| **45 - 50 Years** | **1.4321** | **1.0689** | **1.9188** | **0.0161** |
| 50 - 55 Years | 1.1167 | 0.8237 | 1.5139 | 0.4772 |
| 55 - 60 Years | 1.3161 | 0.9831 | 1.7620 | 0.0650 |
| **60 - 65 Years** | **1.5086** | **1.1440** | **1.9894** | **0.0036** |
| 65 - 70 Years | 1.1865 | 0.8569 | 1.6430 | 0.3030 |
| **70 - 75 Years** | **2.8072** | **2.1498** | **3.6657** | **<0.0001** |
| **75 - 80 Years** | **3.6831** | **2.8141** | **4.8205** | **<0.0001** |
| **≥80 Years** | **5.9490** | **4.6114** | **7.6747** | **<0.0001** |
| Male (vs. Female) | 0.9973 | 0.8635 | 1.1517 | 0.9702 |
| **Concussion** | **0.5508** | **0.3878** | **0.7824** | **0.0009** |
| Traumatic cerebral edema | 1.0180 | 0.8592 | 1.2063 | 0.8364 |
| **Diffuse traumatic brain injury** | **1.4468** | **1.2484** | **1.6767** | **<0.0001** |
| Epidural hemorrhage | 0.9991 | 0.7910 | 1.2619 | 0.9937 |
| **Traumatic subdural hemorrhage** | **1.3419** | **1.1475** | **1.5693** | **0.0002** |
| Traumatic subarachnoid hemorrhage | 1.0363 | 0.8856 | 1.2128 | 0.6562 |
| **Traumatic brain compression and herniation** | **0.6430** | **0.4925** | **0.8394** | **0.0012** |
| Unspecified focal traumatic brain injury | 0.8778 | 0.6808 | 1.1319 | 0.3150 |
| Contusion and laceration of right cerebrum | 1.0703 | 0.7592 | 1.5088 | 0.6983 |
| Contusion and laceration of left cerebrum | 1.2561 | 0.9059 | 1.7416 | 0.1716 |
| Traumatic hemorrhage of right cerebrum | 1.1781 | 0.8979 | 1.5458 | 0.2368 |
| Traumatic hemorrhage of left cerebrum | 0.7981 | 0.5920 | 1.0761 | 0.1391 |
| Contusion, laceration, or hemorrhage of brainstem | 0.8032 | 0.4416 | 1.4611 | 0.4728 |
| Fracture of skull and facial bones | 1.0545 | 0.9064 | 1.2267 | 0.4920 |
| Dislocation/sprain of joints/ligaments at neck level | 1.3105 | 0.9508 | 1.8064 | 0.0986 |
| **Injury of nerves and spinal cord at neck level** | **0.5872** | **0.3927** | **0.8781** | **0.0095** |
| Blood Pressure, Systolic (<90 mmHg vs. ≥90 mmHg) | 0.9949 | 0.8395 | 1.1791 | 0.9533 |
| Nontraumatic subarachnoid hemorrhage | 0.9372 | 0.7936 | 1.1067 | 0.4444 |
| Nontraumatic intracerebral hemorrhage | 0.9790 | 0.8128 | 1.1792 | 0.8232 |
| Unspecified nontraumatic intracranial hemorrhage | 0.9471 | 0.8072 | 1.1112 | 0.5047 |
| Cerebral infarction | 1.1798 | 0.9417 | 1.4781 | 0.1505 |
| Physiological Systems / Performance / Respiratory | 0.9685 | 0.8361 | 1.1217 | 0.6691 |
| Craniectomy or Craniotomy Procedures | 0.9722 | 0.7691 | 1.2289 | 0.8134 |
| Twist Drill, Burr Hole(s), or Trephine Procedures | 1.2054 | 0.9319 | 1.5591 | 0.1548 |
| **Routine Electroencephalography (EEG) Procedures** | **1.9355** | **1.4374** | **2.6062** | **<0.0001** |
| Sympathomimetics | 0.8742 | 0.7476 | 1.0222 | 0.0920 |
| Transfusion, blood or blood components | 0.9955 | 0.8117 | 1.2209 | 0.9653 |
| Emergency Department Services | 1.0362 | 0.9055 | 1.1858 | 0.6051 |
| **Disorientation, amnesia** | **1.3044** | **1.1307** | **1.5048** | **0.0003** |
| Stupor | 0.9748 | 0.6154 | 1.5439 | 0.9133 |
| Malaise and fatigue | 1.0569 | 0.8077 | 1.3829 | 0.6869 |
| **Abnormal movements, gait disturbance** | **1.7431** | **1.4639** | **2.0755** | **<0.0001** |
| Alcohol related disorders | 1.1284 | 0.9598 | 1.3266 | 0.1434 |

# Supplemental Table 6. GCS 13-15, 100 mg Gabapentin, Risk of Durable Cognitive Impairment by 2 Years

| **Covariate** | **Hazard Ratio** | **95 % CI Lower** | **95 % CI Upper** | **P > \|z\|** |
| --- | --- | --- | --- | --- |
| **Gabapentin 100 mg (vs. no gabapentin)** | **0.5833** | **0.3709** | **0.9174** | **0.0196** |
| Age at Index [REF = patients 18-30 years old] |  |  |  |  |
| 30 - 35 Years | 1.4826 | 0.9479 | 2.3189 | 0.0844 |
| **35 - 40 Years** | **1.9315** | **1.2577** | **2.9664** | **0.0026** |
| **40 - 45 Years** | **1.9449** | **1.2495** | **3.0271** | **0.0032** |
| **45 - 50 Years** | **2.6482** | **1.7763** | **3.9482** | **<0.0001** |
| **50 - 55 Years** | **3.2785** | **2.2850** | **4.7042** | **<0.0001** |
| **55 - 60 Years** | **4.4663** | **3.2129** | **6.2087** | **<0.0001** |
| **60 - 65 Years** | **6.5540** | **4.8100** | **8.9303** | **<0.0001** |
| **65 - 70 Years** | **10.0656** | **7.5013** | **13.5065** | **<0.0001** |
| **70 - 75 Years** | **14.1315** | **10.6029** | **18.8345** | **<0.0001** |
| **75 - 80 Years** | **20.0297** | **15.0937** | **26.5797** | **<0.0001** |
| **≥80 Years** | **34.9915** | **26.5318** | **46.1487** | **<0.0001** |
| Male (vs. Female) | 1.0305 | 0.9450 | 1.1237 | 0.4967 |
| **Concussion** | **0.6544** | **0.5633** | **0.7603** | **<0.0001** |
| **Traumatic cerebral edema** | **1.2173** | **1.0325** | **1.4353** | **0.0193** |
| Diffuse traumatic brain injury | 1.1339 | 0.9779 | 1.3146 | 0.0960 |
| Epidural hemorrhage | 1.0853 | 0.8808 | 1.3371 | 0.4423 |
| **Traumatic subdural hemorrhage** | **1.1884** | **1.0745** | **1.3142** | **0.0008** |
| Traumatic subarachnoid hemorrhage | 1.0929 | 0.9791 | 1.2199 | 0.1133 |
| Traumatic brain compression and herniation | 0.9392 | 0.7527 | 1.1719 | 0.5787 |
| Unspecified focal traumatic brain injury | 1.1088 | 0.9118 | 1.3484 | 0.3008 |
| Contusion and laceration of right cerebrum | 0.9683 | 0.7237 | 1.2956 | 0.8283 |
| Contusion and laceration of left cerebrum | 1.1292 | 0.8355 | 1.5263 | 0.4292 |
| **Traumatic hemorrhage of right cerebrum** | **1.2247** | **1.0016** | **1.4974** | **0.0482** |
| Traumatic hemorrhage of left cerebrum | 1.1362 | 0.9162 | 1.4089 | 0.2448 |
| Contusion, laceration, or hemorrhage of brainstem | 1.0167 | 0.5735 | 1.8022 | 0.9549 |
| Fracture of skull and facial bones | 0.9382 | 0.8444 | 1.0424 | 0.2350 |
| Dislocation/sprain of joints/ligaments at neck level | 1.1110 | 0.8008 | 1.5413 | 0.5285 |
| **Injury of nerves and spinal cord at neck level** | **0.6953** | **0.4856** | **0.9955** | **0.0472** |
| Blood Pressure, Systolic (<90 mmHg vs. ≥90 mmHg) | 1.0519 | 0.8639 | 1.2807 | 0.6147 |
| Nontraumatic subarachnoid hemorrhage | 1.0645 | 0.9448 | 1.1993 | 0.3044 |
| Nontraumatic intracerebral hemorrhage | 1.1941 | 1.0412 | 1.3696 | 0.0112 |
| Unspecified nontraumatic intracranial hemorrhage | 1.0686 | 0.9630 | 1.1858 | 0.2117 |
| **Cerebral infarction** | **1.2198** | **1.0111** | **1.4716** | **0.0380** |
| Physiological Systems / Performance / Respiratory | 1.1246 | 0.9291 | 1.3612 | 0.2280 |
| Craniectomy or Craniotomy Procedures | 1.0787 | 0.7947 | 1.4642 | 0.6269 |
| Twist Drill, Burr Hole(s), or Trephine Procedures | 0.9772 | 0.6278 | 1.5209 | 0.9185 |
| **Routine Electroencephalography (EEG) Procedures** | **1.5296** | **1.0570** | **2.2136** | **0.0242** |
| Sympathomimetics | 0.9292 | 0.8138 | 1.0610 | 0.2779 |
| Transfusion, blood or blood components | 0.9151 | 0.7151 | 1.1710 | 0.4807 |
| Emergency Department Services | 0.9638 | 0.8848 | 1.0498 | 0.3980 |
| **Disorientation, amnesia** | **1.7849** | **1.6131** | **1.9749** | **<0.0001** |
| Stupor | 0.8725 | 0.5599 | 1.3595 | 0.5467 |
| Malaise and fatigue | 0.9343 | 0.7842 | 1.1130 | 0.4466 |
| **Abnormal movements, gait disturbance** | **1.5744** | **1.4151** | **1.7516** | **<0.0001** |
| Alcohol related disorders | 1.1222 | 0.9869 | 1.2760 | 0.0786 |

# Supplemental Table 7. GCS 13-15, 300 mg Gabapentin, Risk of Durable Cognitive Impairment by 2 Years

| **Covariate** | **Hazard Ratio** | **95 % CI Lower** | **95 % CI Upper** | **P > \|z\|** |
| --- | --- | --- | --- | --- |
| Gabapentin 300 mg (vs. no gabapentin) | 0.9274 | 0.6533 | 1.3165 | 0.6733 |
| Age at Index [REF = patients 18-30 years old] |  |  |  |  |
| 30 - 35 Years | 1.5308 | 0.9760 | 2.4008 | 0.0637 |
| **35 - 40 Years** | **1.9892** | **1.2916** | **3.0637** | **0.0018** |
| **40 - 45 Years** | **2.1966** | **1.4262** | **3.3832** | **0.0004** |
| **45 - 50 Years** | **2.8034** | **1.8796** | **4.1812** | **<0.0001** |
| **50 - 55 Years** | **3.4042** | **2.3677** | **4.8944** | **<0.0001** |
| **55 - 60 Years** | **4.6620** | **3.3434** | **6.5006** | **<0.0001** |
| **60 - 65 Years** | **6.8809** | **5.0346** | **9.4044** | **<0.0001** |
| **65 - 70 Years** | **10.5696** | **7.8468** | **14.2372** | **<0.0001** |
| **70 - 75 Years** | **14.6825** | **10.9696** | **19.6520** | **<0.0001** |
| **75 - 80 Years** | **20.9978** | **15.7574** | **27.9811** | **<0.0001** |
| **≥80 Years** | **37.0484** | **27.9711** | **49.0714** | **<0.0001** |
| Male (vs. Female) | 1.0328 | 0.9474 | 1.1260 | 0.4637 |
| **Concussion** | **0.6598** | **0.5683** | **0.7659** | **<0.0001** |
| **Traumatic cerebral edema** | **1.2077** | **1.0244** | **1.4238** | **0.0247** |
| Diffuse traumatic brain injury | 1.1511 | 0.9938 | 1.3334 | 0.0605 |
| Epidural hemorrhage | 1.0773 | 0.8727 | 1.3299 | 0.4883 |
| Traumatic subdural hemorrhage | 1.1805 | 1.0678 | 1.3050 | 0.0012 |
| Traumatic subarachnoid hemorrhage | 1.1037 | 0.9895 | 1.2312 | 0.0767 |
| Traumatic brain compression and herniation | 0.9606 | 0.7707 | 1.1973 | 0.7204 |
| Unspecified focal traumatic brain injury | 1.1205 | 0.9228 | 1.3605 | 0.2506 |
| Contusion and laceration of right cerebrum | 0.9666 | 0.7239 | 1.2906 | 0.8177 |
| Contusion and laceration of left cerebrum | 1.2065 | 0.9027 | 1.6126 | 0.2047 |
| Traumatic hemorrhage of right cerebrum | 1.2169 | 0.9960 | 1.4869 | 0.0548 |
| Traumatic hemorrhage of left cerebrum | 1.1665 | 0.9433 | 1.4425 | 0.1551 |
| Contusion, laceration, or hemorrhage of brainstem | 1.0280 | 0.5798 | 1.8225 | 0.9248 |
| Fracture of skull and facial bones | 0.9452 | 0.8512 | 1.0495 | 0.2911 |
| Dislocation/sprain of joints/ligaments at neck level | 1.0689 | 0.7673 | 1.4892 | 0.6935 |
| **Injury of nerves and spinal cord at neck level** | **0.6916** | **0.4830** | **0.9905** | **0.0442** |
| Blood Pressure, Systolic (<90 mmHg vs. ≥90 mmHg) | 1.0746 | 0.8845 | 1.3056 | 0.4687 |
| Nontraumatic subarachnoid hemorrhage | 1.0534 | 0.9354 | 1.1862 | 0.3910 |
| **Nontraumatic intracerebral hemorrhage** | **1.1910** | **1.0389** | **1.3655** | **0.0122** |
| Unspecified nontraumatic intracranial hemorrhage | 1.0730 | 0.9671 | 1.1906 | 0.1838 |
| Cerebral infarction | 1.1862 | 0.9816 | 1.4336 | 0.0771 |
| Physiological Systems / Performance / Respiratory | 1.1103 | 0.9169 | 1.3445 | 0.2841 |
| Craniectomy or Craniotomy Procedures | 1.0641 | 0.7842 | 1.4438 | 0.6900 |
| Twist Drill, Burr Hole(s), or Trephine Procedures | 0.9760 | 0.6272 | 1.5190 | 0.9144 |
| **Routine Electroencephalography (EEG) Procedures** | **1.7405** | **1.2301** | **2.4626** | **0.0018** |
| Sympathomimetics | 0.9253 | 0.8105 | 1.0565 | 0.2513 |
| Transfusion, blood or blood components | 0.9116 | 0.7124 | 1.1666 | 0.4621 |
| Emergency Department Services | 0.9690 | 0.8899 | 1.0552 | 0.4693 |
| **Disorientation, amnesia** | **1.7544** | **1.5858** | **1.9410** | **<0.0001** |
| Stupor | 0.8836 | 0.5671 | 1.3768 | 0.5845 |
| Malaise and fatigue | 0.9233 | 0.7747 | 1.1005 | 0.3731 |
| **Abnormal movements, gait disturbance** | **1.5952** | **1.4346** | **1.7738** | **<0.0001** |
| Alcohol related disorders | 1.1347 | 0.9990 | 1.2888 | 0.0518 |

# Supplemental Table 8. All GCS, Risk of Mortality by 2 Years

| **Covariate** | **Hazard Ratio** | **95 % CI Lower** | **95 % CI Upper** | **P > \|z\|** |
| --- | --- | --- | --- | --- |
| **Gabapentin (vs. no gabapentin)** | **0.6682** | **0.5562** | **0.8028** | **<0.0001** |
| **GCS 13-15** | **0.3151** | **0.2737** | **0.3627** | **<0.0001** |
| **GCS 9-12** | **0.8300** | **0.7341** | **0.9384** | **0.0029** |
| **GCS 3-8** | **2.8464** | **2.4798** | **3.2673** | **<0.0001** |
| Age at Index [REF = patients 18-30 years old] |  |  |  |  |
| **30 - 35 Years** | **1.1974** | **1.0609** | **1.3515** | **0.0035** |
| **35 - 40 Years** | **1.3116** | **1.1542** | **1.4904** | **<0.0001** |
| **40 - 45 Years** | **1.3050** | **1.1426** | **1.4905** | **0.0001** |
| **45 - 50 Years** | **1.4971** | **1.3169** | **1.7019** | **<0.0001** |
| **50 - 55 Years** | **1.7198** | **1.5302** | **1.9329** | **<0.0001** |
| **55 - 60 Years** | **2.1941** | **1.9703** | **2.4434** | **<0.0001** |
| **60 - 65 Years** | **2.4685** | **2.2266** | **2.7366** | **<0.0001** |
| **65 - 70 Years** | **3.0241** | **2.7314** | **3.3481** | **<0.0001** |
| **70 - 75 Years** | **3.6192** | **3.2716** | **4.0037** | **<0.0001** |
| **75 - 80 Years** | **4.7687** | **4.3134** | **5.2721** | **<0.0001** |
| **≥80 Years** | **6.2541** | **5.6787** | **6.8879** | **<0.0001** |
| **Male (vs. Female)** | **1.1015** | **1.0463** | **1.1597** | **0.0002** |
| **Concussion** | **1.5695** | **1.4750** | **1.6701** | **<0.0001** |
| **Traumatic cerebral edema** | **0.9275** | **0.8707** | **0.9880** | **0.0196** |
| **Diffuse traumatic brain injury** | **0.7973** | **0.7216** | **0.8809** | **<0.0001** |
| **Epidural hemorrhage** | **1.0580** | **1.0011** | **1.1181** | **0.0458** |
| **Traumatic subdural hemorrhage** | **0.8886** | **0.8399** | **0.9401** | **<0.0001** |
| **Traumatic subarachnoid hemorrhage** | **1.2562** | **1.1591** | **1.3615** | **<0.0001** |
| Traumatic brain compression and herniation | 1.0840 | 0.9881 | 1.1892 | 0.0880 |
| **Unspecified focal traumatic brain injury** | **0.7663** | **0.6530** | **0.8992** | **0.0011** |
| **Contusion and laceration of right cerebrum** | **0.7182** | **0.6104** | **0.8452** | **0.0001** |
| Contusion and laceration of left cerebrum | 1.0193 | 0.9177 | 1.1323 | 0.7209 |
| **Traumatic hemorrhage of right cerebrum** | **0.8462** | **0.7590** | **0.9433** | **0.0026** |
| **Traumatic hemorrhage of left cerebrum** | **1.8113** | **1.5239** | **2.1528** | **<0.0001** |
| **Contusion, laceration, or hemorrhage of brainstem** | **0.9369** | **0.8863** | **0.9904** | **0.0214** |
| **Fracture of skull and facial bones** | **0.6475** | **0.5527** | **0.7586** | **<0.0001** |
| Dislocation/sprain of joints/ligaments at neck level | 0.9038 | 0.7874 | 1.0376 | 0.1510 |
| **Injury of nerves and spinal cord at neck level** | **1.5695** | **1.4750** | **1.6701** | **<0.0001** |
| **Blood Pressure, Systolic (<90 mmHg vs. ≥90 mmHg)** | **1.3067** | **1.2308** | **1.3873** | **<0.0001** |
| **Nontraumatic subarachnoid hemorrhage** | **0.9153** | **0.8593** | **0.9750** | **0.0060** |
| **Nontraumatic intracerebral hemorrhage** | **1.2801** | **1.1975** | **1.3684** | **<0.0001** |
| **Unspecified nontraumatic intracranial hemorrhage** | **1.0779** | **1.0174** | **1.1420** | **0.0109** |
| **Cerebral infarction** | **1.1025** | **1.0124** | **1.2007** | **0.0249** |
| **Physiological Systems / Performance / Respiratory** | **1.1268** | **1.0602** | **1.1977** | **0.0001** |
| **Craniectomy or Craniotomy Procedures** | **0.7313** | **0.6691** | **0.7994** | **<0.0001** |
| Twist Drill, Burr Hole(s), or Trephine Procedures | 0.9154 | 0.8200 | 1.0220 | 0.1158 |
| **Routine Electroencephalography (EEG) Procedures** | **0.6813** | **0.5760** | **0.8059** | **<0.0001** |
| **Sympathomimetics** | **1.4052** | **1.3279** | **1.4871** | **<0.0001** |
| **Transfusion, blood or blood components** | **1.5124** | **1.4120** | **1.6200** | **<0.0001** |
| **Emergency Department Services** | **0.8359** | **0.7951** | **0.8788** | **<0.0001** |
| **Disorientation, amnesia** | **0.6630** | **0.6221** | **0.7066** | **<0.0001** |
| Stupor | 0.8470 | 0.6810 | 1.0533 | 0.1355 |
| **Malaise and fatigue** | **1.1324** | **1.0168** | **1.2612** | **0.0237** |
| **Abnormal movements, gait disturbance** | **0.9107** | **0.8436** | **0.9831** | **0.0166** |
| **Alcohol related disorders** | **0.6098** | **0.5681** | **0.6545** | **<0.0001** |

# Supplemental Table 9. GCS 13-15, Risk of Mortality by 2 Years

| **Covariate** | **Hazard Ratio** | **95 % CI Lower** | **95 % CI Upper** | **P > \|z\|** |
| --- | --- | --- | --- | --- |
| Gabapentin (vs. no gabapentin) | 0.9065 | 0.7046 | 1.1661 | 0.4447 |
| Age at Index [REF = patients 18-30 years old] |  |  |  |  |
| 30 - 35 Years | 1.2447 | 0.7039 | 2.2009 | 0.4517 |
| **35 - 40 Years** | **2.2034** | **1.3483** | **3.6010** | **0.0016** |
| **40 - 45 Years** | **2.5518** | **1.5695** | **4.1489** | **0.0002** |
| **45 - 50 Years** | **3.2309** | **2.0641** | **5.0574** | **<0.0001** |
| **50 - 55 Years** | **4.4810** | **3.0004** | **6.6921** | **<0.0001** |
| **55 - 60 Years** | **6.6458** | **4.5908** | **9.6206** | **<0.0001** |
| **60 - 65 Years** | **8.1017** | **5.6599** | **11.5970** | **<0.0001** |
| **65 - 70 Years** | **10.1173** | **7.1115** | **14.3934** | **<0.0001** |
| **70 - 75 Years** | **13.3762** | **9.4484** | **18.9369** | **<0.0001** |
| **75 - 80 Years** | **19.3836** | **13.7764** | **27.2729** | **<0.0001** |
| **≥80 Years** | **30.4481** | **21.7453** | **42.6338** | **<0.0001** |
| **Male (vs. Female)** | **1.3559** | **1.2195** | **1.5075** | **<0.0001** |
| **Concussion** | **0.6358** | **0.5302** | **0.7624** | **<0.0001** |
| **Traumatic cerebral edema** | **1.3966** | **1.1692** | **1.6682** | **0.0002** |
| Diffuse traumatic brain injury | 1.1456 | 0.9664 | 1.3580 | 0.1173 |
| Epidural hemorrhage | 0.8641 | 0.6790 | 1.0996 | 0.2349 |
| **Traumatic subdural hemorrhage** | **1.1563** | **1.0256** | **1.3037** | **0.0176** |
| Traumatic subarachnoid hemorrhage | 1.0217 | 0.8961 | 1.1649 | 0.7483 |
| Traumatic brain compression and herniation | 1.1836 | 0.9316 | 1.5038 | 0.1675 |
| Unspecified focal traumatic brain injury | 1.0547 | 0.8362 | 1.3304 | 0.6528 |
| Contusion and laceration of right cerebrum | 0.8038 | 0.5652 | 1.1432 | 0.2242 |
| Contusion and laceration of left cerebrum | 0.7182 | 0.4759 | 1.0840 | 0.1150 |
| Traumatic hemorrhage of right cerebrum | 1.2613 | 0.9892 | 1.6083 | 0.0612 |
| Traumatic hemorrhage of left cerebrum | 0.8546 | 0.6405 | 1.1402 | 0.2854 |
| Contusion, laceration, or hemorrhage of brainstem | 1.0345 | 0.5337 | 2.0053 | 0.9199 |
| **Fracture of skull and facial bones** | **0.8361** | **0.7368** | **0.9488** | **0.0055** |
| Dislocation/sprain of joints/ligaments at neck level | 1.0417 | 0.7439 | 1.4585 | 0.8121 |
| Injury of nerves and spinal cord at neck level | 1.2891 | 0.9547 | 1.7408 | 0.0975 |
| **Blood Pressure, Systolic (<90 mmHg vs. ≥90 mmHg)** | **1.3860** | **1.1568** | **1.6607** | **0.0004** |
| Nontraumatic subarachnoid hemorrhage | 1.0676 | 0.9244 | 1.2329 | 0.3733 |
| **Nontraumatic intracerebral hemorrhage** | **1.2730** | **1.0855** | **1.4928** | **0.0030** |
| **Unspecified nontraumatic intracranial hemorrhage** | **1.2305** | **1.0903** | **1.3886** | **0.0008** |
| **Cerebral infarction** | **1.7272** | **1.4242** | **2.0947** | **<0.0001** |
| **Physiological Systems / Performance / Respiratory** | **3.1729** | **2.7044** | **3.7225** | **<0.0001** |
| Craniectomy or Craniotomy Procedures | 1.2566 | 0.9457 | 1.6696 | 0.1152 |
| Twist Drill, Burr Hole(s), or Trephine Procedures | 0.9246 | 0.5985 | 1.4285 | 0.7240 |
| Routine Electroencephalography (EEG) Procedures | 0.8691 | 0.5191 | 1.4550 | 0.5936 |
| Sympathomimetics | 0.9440 | 0.8171 | 1.0905 | 0.4334 |
| **Transfusion, blood or blood components** | **1.5966** | **1.3040** | **1.9547** | **<0.0001** |
| **Emergency Department Services** | **0.6822** | **0.6157** | **0.7558** | **<0.0001** |
| **Disorientation, amnesia** | **1.1640** | **1.0256** | **1.3211** | **0.0187** |
| Stupor | 0.8093 | 0.4841 | 1.3528 | 0.4195 |
| **Malaise and fatigue** | **1.4497** | **1.2145** | **1.7306** | **<0.0001** |
| **Abnormal movements, gait disturbance** | **1.2634** | **1.1084** | **1.4400** | **0.0005** |
| **Alcohol related disorders** | **1.1947** | **1.0351** | **1.3788** | **0.0150** |

# Supplemental Table 10. GCS 3-8, Risk of Mortality by 2 Years

| **Covariate** | **Hazard Ratio** | **95 % CI Lower** | **95 % CI Upper** | **P > \|z\|** |
| --- | --- | --- | --- | --- |
| **Gabapentin (vs. no gabapentin)** | **0.5375** | **0.4018** | **0.7191** | **<0.0001** |
| Age at Index [REF = patients 18-30 years old] |  |  |  |  |
| **30 - 35 Years** | **1.1951** | **1.0543** | **1.3547** | **0.0053** |
| **35 - 40 Years** | **1.3036** | **1.1383** | **1.4931** | **0.0001** |
| **40 - 45 Years** | **1.2875** | **1.1174** | **1.4835** | **0.0005** |
| **45 - 50 Years** | **1.4367** | **1.2507** | **1.6503** | **<0.0001** |
| **50 - 55 Years** | **1.5834** | **1.3932** | **1.7996** | **<0.0001** |
| **55 - 60 Years** | **1.8321** | **1.6228** | **2.0683** | **<0.0001** |
| **60 - 65 Years** | **2.0444** | **1.8192** | **2.2974** | **<0.0001** |
| **65 - 70 Years** | **2.4561** | **2.1838** | **2.7623** | **<0.0001** |
| **70 - 75 Years** | **2.7515** | **2.4445** | **3.0969** | **<0.0001** |
| **75 - 80 Years** | **3.3306** | **2.9451** | **3.7665** | **<0.0001** |
| **≥80 Years** | **3.7495** | **3.3153** | **4.2406** | **<0.0001** |
| Male (vs. Female) | 1.0183 | 0.9569 | 1.0837 | 0.5670 |
| **Concussion** | **0.2659** | **0.2148** | **0.3292** | **<0.0001** |
| **Traumatic cerebral edema** | **1.5288** | **1.4269** | **1.6381** | **<0.0001** |
| **Diffuse traumatic brain injury** | **0.8806** | **0.8209** | **0.9448** | **0.0004** |
| **Epidural hemorrhage** | **0.7394** | **0.6582** | **0.8307** | **<0.0001** |
| Traumatic subdural hemorrhage | 0.9949 | 0.9318 | 1.0624 | 0.8794 |
| **Traumatic subarachnoid hemorrhage** | **0.8307** | **0.7777** | **0.8873** | **<0.0001** |
| **Traumatic brain compression and herniation** | **1.2495** | **1.1422** | **1.3668** | **<0.0001** |
| Unspecified focal traumatic brain injury | 1.1121 | 1.0002 | 1.2365 | 0.0495 |
| **Contusion and laceration of right cerebrum** | **0.7980** | **0.6624** | **0.9613** | **0.0175** |
| **Contusion and laceration of left cerebrum** | **0.6752** | **0.5562** | **0.8198** | **0.0001** |
| Traumatic hemorrhage of right cerebrum | 0.9885 | 0.8709 | 1.1220 | 0.8583 |
| **Traumatic hemorrhage of left cerebrum** | **0.8082** | **0.7107** | **0.9190** | **0.0012** |
| **Contusion, laceration, or hemorrhage of brainstem** | **1.8575** | **1.5454** | **2.2326** | **<0.0001** |
| **Fracture of skull and facial bones** | **0.9324** | **0.8736** | **0.9952** | **0.0353** |
| **Dislocation/sprain of joints/ligaments at neck level** | **0.6150** | **0.5113** | **0.7397** | **<0.0001** |
| **Injury of nerves and spinal cord at neck level** | **0.7871** | **0.6685** | **0.9269** | **0.0041** |
| **Blood Pressure, Systolic (<90 mmHg vs. ≥90 mmHg)** | **1.3003** | **1.2179** | **1.3883** | **<0.0001** |
| **Nontraumatic subarachnoid hemorrhage** | **0.8989** | **0.8339** | **0.9690** | **0.0054** |
| **Nontraumatic intracerebral hemorrhage** | **1.2289** | **1.1368** | **1.3284** | **<0.0001** |
| Unspecified nontraumatic intracranial hemorrhage | 1.0157 | 0.9469 | 1.0895 | 0.6633 |
| Cerebral infarction | 0.9770 | 0.8827 | 1.0813 | 0.6525 |
| Physiological Systems / Performance / Respiratory | 0.9641 | 0.9037 | 1.0285 | 0.2680 |
| **Craniectomy or Craniotomy Procedures** | **0.7240** | **0.6565** | **0.7986** | **<0.0001** |
| Twist Drill, Burr Hole(s), or Trephine Procedures | 0.8964 | 0.7967 | 1.0087 | 0.0694 |
| **Routine Electroencephalography (EEG) Procedures** | **0.6532** | **0.5385** | **0.7924** | **<0.0001** |
| **Sympathomimetics** | **1.4967** | **1.4024** | **1.5972** | **<0.0001** |
| **Transfusion, blood or blood components** | **1.3848** | **1.2844** | **1.4931** | **<0.0001** |
| **Emergency Department Services** | **0.9382** | **0.8837** | **0.9960** | **0.0367** |
| **Disorientation, amnesia** | **0.5336** | **0.4931** | **0.5775** | **<0.0001** |
| Stupor | 0.8267 | 0.6384 | 1.0705 | 0.1490 |
| Malaise and fatigue | 0.9335 | 0.8064 | 1.0808 | 0.3573 |
| **Abnormal movements, gait disturbance** | **0.7143** | **0.6423** | **0.7944** | **<0.0001** |
| **Alcohol related disorders** | **0.5192** | **0.4764** | **0.5658** | **<0.0001** |

# Supplemental Table 11. All GCS, Risk of Durable Cognitive Impairment by 2 Years, Controlling for Levetiracetam

| **Covariate** | **Hazard Ratio** | **95 % CI Lower** | **95 % CI Upper** | **P > \|z\|** |
| --- | --- | --- | --- | --- |
| Gabapentin (vs. no gabapentin) | 0.8521 | 0.7016 | 1.0347 | 0.1062 |
| Levetiracetam (vs. no levetiracetam) | 0.9966 | 0.9248 | 1.0738 | 0.9279 |
| GCS 13-15 | 0.9609 | 0.8161 | 1.1315 | 0.6326 |
| **GCS 9-12** | **1.4231** | **1.2216** | **1.6578** | **<0.0001** |
| GCS 3-8 | 1.1180 | 0.9457 | 1.3215 | 0.1914 |
| Age at Index [REF = patients 18-30 years old] |  |  |  |  |
| 30 - 35 Years | 1.0636 | 0.8440 | 1.3402 | 0.6015 |
| 35 - 40 Years | 1.0169 | 0.7897 | 1.3095 | 0.8965 |
| **40 - 45 Years** | **1.2978** | **1.0233** | **1.6461** | **0.0316** |
| **45 - 50 Years** | **1.6186** | **1.3018** | **2.0124** | **<0.0001** |
| **50 - 55 Years** | **1.5333** | **1.2395** | **1.8968** | **0.0001** |
| **55 - 60 Years** | **2.0184** | **1.6700** | **2.4396** | **<0.0001** |
| **60 - 65 Years** | **2.6204** | **2.1990** | **3.1224** | **<0.0001** |
| **65 - 70 Years** | **3.5476** | **3.0052** | **4.1878** | **<0.0001** |
| **70 - 75 Years** | **5.5175** | **4.7171** | **6.4538** | **<0.0001** |
| **75 - 80 Years** | **7.7230** | **6.6276** | **8.9994** | **<0.0001** |
| **≥80 Years** | **13.2295** | **11.4397** | **15.2994** | **<0.0001** |
| Male (vs. Female) | 1.0052 | 0.9377 | 1.0776 | 0.8832 |
| **Concussion** | **0.6084** | **0.5350** | **0.6918** | **<0.0001** |
| Traumatic cerebral edema | 1.0785 | 0.9637 | 1.2069 | 0.1880 |
| **Diffuse traumatic brain injury** | **1.3129** | **1.1902** | **1.4484** | **<0.0001** |
| Epidural hemorrhage | 1.0826 | 0.9358 | 1.2524 | 0.2859 |
| **Traumatic subdural hemorrhage** | **1.2572** | **1.1603** | **1.3622** | **<0.0001** |
| **Traumatic subarachnoid hemorrhage** | **1.0972** | **1.0086** | **1.1935** | **0.0308** |
| **Traumatic brain compression and herniation** | **0.7817** | **0.6665** | **0.9168** | **0.0025** |
| Unspecified focal traumatic brain injury | 1.0348 | 0.8954 | 1.1959 | 0.6433 |
| Contusion and laceration of right cerebrum | 0.9689 | 0.7832 | 1.1986 | 0.7708 |
| **Contusion and laceration of left cerebrum** | **1.2460** | **1.0193** | **1.5233** | **0.0318** |
| **Traumatic hemorrhage of right cerebrum** | **1.1988** | **1.0296** | **1.3958** | **0.0195** |
| Traumatic hemorrhage of left cerebrum | 0.9477 | 0.8047 | 1.1161 | 0.5194 |
| Contusion, laceration, or hemorrhage of brainstem | 0.9146 | 0.6202 | 1.3489 | 0.6527 |
| Fracture of skull and facial bones | 0.9812 | 0.9053 | 1.0633 | 0.6429 |
| Dislocation/sprain of joints/ligaments at neck level | 1.1638 | 0.9343 | 1.4497 | 0.1759 |
| **Injury of nerves and spinal cord at neck level** | **0.6732** | **0.5250** | **0.8633** | **0.0018** |
| **Blood Pressure, Systolic (<90 mmHg vs. ≥90 mmHg)** | 1.0297 | 0.9141 | 1.1599 | 0.6303 |
| Nontraumatic subarachnoid hemorrhage | 1.0258 | 0.9375 | 1.1224 | 0.5786 |
| Nontraumatic intracerebral hemorrhage | 1.0597 | 0.9551 | 1.1758 | 0.2740 |
| Unspecified nontraumatic intracranial hemorrhage | 1.0131 | 0.9335 | 1.0995 | 0.7554 |
| **Cerebral infarction** | **1.1644** | **1.0167** | **1.3336** | **0.0279** |
| Physiological Systems / Performance / Respiratory | 1.0461 | 0.9407 | 1.1632 | 0.4056 |
| Craniectomy or Craniotomy Procedures | 0.9631 | 0.8080 | 1.1480 | 0.6751 |
| Twist Drill, Burr Hole(s), or Trephine Procedures | 1.2068 | 0.9728 | 1.4970 | 0.0874 |
| **Routine Electroencephalography (EEG) Procedures** | **1.6693** | **1.3413** | **2.0775** | **<0.0001** |
| **Sympathomimetics** | **0.8679** | **0.7884** | **0.9554** | **0.0038** |
| Transfusion, blood or blood components | 0.9579 | 0.8283 | 1.1076 | 0.5613 |
| Emergency Department Services | 0.9532 | 0.8906 | 1.0202 | 0.1669 |
| **Disorientation, amnesia** | **1.5916** | **1.4731** | **1.7198** | **<0.0001** |
| Stupor | 0.9421 | 0.7046 | 1.2596 | 0.6873 |
| Malaise and fatigue | 1.0098 | 0.8812 | 1.1572 | 0.8881 |
| **Abnormal movements, gait disturbance** | **1.6800** | **1.5428** | **1.8295** | **<0.0001** |
| **Alcohol related disorders** | **1.1598** | **1.0563** | **1.2733** | **0.0019** |

# Supplemental Table 12. All GCS, Risk of Durable Cognitive Impairment by 2 Years, Controlling for Levetiracetam, Seizures, and Epilepsy

| **Covariate** | **Hazard Ratio** | **95 % CI Lower** | **95 % CI Upper** | **P > \|z\|** |
| --- | --- | --- | --- | --- |
| Gabapentin (vs. no gabapentin) | 0.8537 | 0.7030 | 1.0367 | 0.1105 |
| Levetiracetam (vs. no levetiracetam) | 0.9718 | 0.9013 | 1.0477 | 0.4559 |
| GCS 13-15 | 0.9571 | 0.8131 | 1.1267 | 0.5985 |
| **GCS 9-12** | **1.3970** | **1.1990** | **1.6277** | **<0.0001** |
| GCS 3-8 | 1.0944 | 0.9258 | 1.2936 | 0.2906 |
| Age at Index [REF = patients 18-30 years old] |  |  |  |  |
| 30 - 35 Years | 1.0628 | 0.8434 | 1.3393 | 0.6056 |
| 35 - 40 Years | 1.0123 | 0.7861 | 1.3036 | 0.9244 |
| **40 - 45 Years** | **1.2831** | **1.0116** | **1.6274** | **0.0399** |
| **45 - 50 Years** | **1.6108** | **1.2955** | **2.0027** | **<0.0001** |
| **50 - 55 Years** | **1.5197** | **1.2285** | **1.8800** | **0.0001** |
| **55 - 60 Years** | **1.9885** | **1.6451** | **2.4036** | **<0.0001** |
| **60 - 65 Years** | **2.5937** | **2.1766** | **3.0908** | **<0.0001** |
| **65 - 70 Years** | **3.5091** | **2.9725** | **4.1425** | **<0.0001** |
| **70 - 75 Years** | **5.4686** | **4.6753** | **6.3966** | **<0.0001** |
| **75 - 80 Years** | **7.6776** | **6.5887** | **8.9464** | **<0.0001** |
| **≥80 Years** | **13.2520** | **11.4602** | **15.3239** | **<0.0001** |
| Male (vs. Female) | 1.0013 | 0.9340 | 1.0733 | 0.9718 |
| **Concussion** | **0.6089** | **0.5355** | **0.6923** | **<0.0001** |
| Traumatic cerebral edema | 1.0600 | 0.9470 | 1.1866 | 0.3111 |
| **Diffuse traumatic brain injury** | **1.3154** | **1.1925** | **1.4509** | **<0.0001** |
| Epidural hemorrhage | 1.0793 | 0.9330 | 1.2486 | 0.3047 |
| **Traumatic subdural hemorrhage** | **1.2555** | **1.1587** | **1.3604** | **<0.0001** |
| **Traumatic subarachnoid hemorrhage** | **1.1043** | **1.0151** | **1.2012** | **0.0209** |
| **Traumatic brain compression and herniation** | **0.7779** | **0.6632** | **0.9125** | **0.0020** |
| Unspecified focal traumatic brain injury | 1.0331 | 0.8939 | 1.1939 | 0.6593 |
| Contusion and laceration of right cerebrum | 0.9675 | 0.7818 | 1.1972 | 0.7608 |
| **Contusion and laceration of left cerebrum** | **1.2312** | **1.0068** | **1.5055** | **0.0427** |
| **Traumatic hemorrhage of right cerebrum** | **1.1997** | **1.0305** | **1.3967** | **0.0189** |
| Traumatic hemorrhage of left cerebrum | 0.9496 | 0.8064 | 1.1182 | 0.5351 |
| Contusion, laceration, or hemorrhage of brainstem | 0.9213 | 0.6247 | 1.3586 | 0.6791 |
| Fracture of skull and facial bones | 0.9928 | 0.9161 | 1.0759 | 0.8600 |
| Dislocation/sprain of joints/ligaments at neck level | 1.1701 | 0.9393 | 1.4577 | 0.1612 |
| **Injury of nerves and spinal cord at neck level** | **0.6783** | **0.5288** | **0.8700** | **0.0022** |
| Blood Pressure, Systolic (<90 mmHg vs. ≥90 mmHg) | 1.0237 | 0.9356 | 1.1201 | 0.6102 |
| Nontraumatic subarachnoid hemorrhage | 1.0621 | 0.9573 | 1.1784 | 0.2556 |
| Nontraumatic intracerebral hemorrhage | 1.0077 | 0.9284 | 1.0938 | 0.8542 |
| Unspecified nontraumatic intracranial hemorrhage | 1.1312 | 0.9873 | 1.2961 | 0.0757 |
| Cerebral infarction | 1.0452 | 0.9401 | 1.1620 | 0.4136 |
| Physiological Systems / Performance / Respiratory | 0.9322 | 0.7817 | 1.1116 | 0.4345 |
| Craniectomy or Craniotomy Procedures | 1.2225 | 0.9853 | 1.5168 | 0.0679 |
| **Twist Drill, Burr Hole(s), or Trephine Procedures** | **1.5162** | **1.2148** | **1.8923** | **0.0002** |
| **Routine Electroencephalography (EEG) Procedures** | **0.8698** | **0.7901** | **0.9574** | **0.0044** |
| Sympathomimetics | 0.9518 | 0.8231 | 1.1008 | 0.5057 |
| Transfusion, blood or blood components | 0.9552 | 0.8924 | 1.0224 | 0.1866 |
| **Emergency Department Services** | **1.5774** | **1.4597** | **1.7046** | **<0.0001** |
| Disorientation, amnesia | 0.9206 | 0.6883 | 1.2313 | 0.5771 |
| Stupor | 0.9942 | 0.8674 | 1.1395 | 0.9332 |
| **Malaise and fatigue** | **1.6619** | **1.5260** | **1.8099** | **<0.0001** |
| **Abnormal movements, gait disturbance** | **1.1502** | **1.0476** | **1.2629** | **0.0033** |
| Alcohol related disorders | 1.0237 | 0.9356 | 1.1201 | 0.6102 |
| Convulsions, not elsewhere classified | 1.0853 | 0.9689 | 1.2158 | 0.1574 |
| **Epilepsy and recurrent seizures** | **1.4061** | **1.2418** | **1.5921** | **<0.0001** |

# Supplemental Table 13. GCS 13-15, Risk of Durable Cognitive Impairment by 2 Years, Controlling for Levetiracetam, Seizures, and Epilepsy

| **Covariate** | **Hazard Ratio** | **95 % CI Lower** | **95 % CI Upper** | **P > \|z\|** |
| --- | --- | --- | --- | --- |
| **Gabapentin (vs. no gabapentin)** | **0.7836** | **0.6262** | **0.9804** | **0.0329** |
| Levetiracetam (vs. no levetiracetam) | 0.9210 | 0.8380 | 1.0121 | 0.0874 |
| Age at Index [REF = patients 18-30 years old] |  |  |  |  |
| 30 - 35 Years | 1.4698 | 0.9537 | 2.2653 | 0.0810 |
| **35 - 40 Years** | **1.8377** | **1.2077** | **2.7963** | **0.0045** |
| **40 - 45 Years** | **2.0528** | **1.3541** | **3.1120** | **0.0007** |
| **45 - 50 Years** | **2.5674** | **1.7409** | **3.7862** | **<0.0001** |
| **50 - 55 Years** | **3.1814** | **2.2430** | **4.5123** | **<0.0001** |
| **55 - 60 Years** | **4.2036** | **3.0519** | **5.7899** | **<0.0001** |
| **60 - 65 Years** | **6.1812** | **4.5775** | **8.3466** | **<0.0001** |
| **65 - 70 Years** | **9.7302** | **7.3203** | **12.9335** | **<0.0001** |
| **70 - 75 Years** | **13.6703** | **10.3502** | **18.0553** | **<0.0001** |
| **75 - 80 Years** | **19.1733** | **14.5744** | **25.2235** | **<0.0001** |
| **≥80 Years** | **34.6727** | **26.5268** | **45.3201** | **<0.0001** |
| Male (vs. Female) | 1.0282 | 0.9446 | 1.1191 | 0.5205 |
| **Concussion** | **0.6641** | **0.5735** | **0.7690** | **<0.0001** |
| **Traumatic cerebral edema** | **1.1901** | **1.0117** | **1.3999** | **0.0357** |
| **Diffuse traumatic brain injury** | **1.1593** | **1.0028** | **1.3401** | **0.0457** |
| Epidural hemorrhage | 1.0896 | 0.8889 | 1.3355 | 0.4087 |
| **Traumatic subdural hemorrhage** | **1.1789** | **1.0671** | **1.3024** | **0.0012** |
| **Traumatic subarachnoid hemorrhage** | **1.1206** | **1.0063** | **1.2480** | **0.0381** |
| Traumatic brain compression and herniation | 0.9672 | 0.7801 | 1.1993 | 0.7615 |
| Unspecified focal traumatic brain injury | 1.1605 | 0.9590 | 1.4044 | 0.1261 |
| Contusion and laceration of right cerebrum | 0.9453 | 0.7077 | 1.2626 | 0.7032 |
| Contusion and laceration of left cerebrum | 1.2361 | 0.9318 | 1.6398 | 0.1416 |
| Traumatic hemorrhage of right cerebrum | 1.2134 | 0.9945 | 1.4804 | 0.0567 |
| Traumatic hemorrhage of left cerebrum | 1.1275 | 0.9132 | 1.3920 | 0.2644 |
| Contusion, laceration, or hemorrhage of brainstem | 0.9994 | 0.5638 | 1.7714 | 0.9983 |
| Fracture of skull and facial bones | 0.9578 | 0.8645 | 1.0613 | 0.4106 |
| Dislocation/sprain of joints/ligaments at neck level | 1.0579 | 0.7652 | 1.4625 | 0.7335 |
| Injury of nerves and spinal cord at neck level | 0.7183 | 0.5088 | 1.0140 | 0.0600 |
| Blood Pressure, Systolic (<90 mmHg vs. ≥90 mmHg) | 1.0527 | 0.8688 | 1.2755 | 0.6003 |
| Nontraumatic subarachnoid hemorrhage | 1.0391 | 0.9246 | 1.1678 | 0.5195 |
| **Nontraumatic intracerebral hemorrhage** | **1.1795** | **1.0303** | **1.3504** | **0.0168** |
| Unspecified nontraumatic intracranial hemorrhage | 1.0593 | 0.9559 | 1.1738 | 0.2714 |
| **Cerebral infarction** | **1.2167** | **1.0134** | **1.4609** | **0.0355** |
| Physiological Systems / Performance / Respiratory | 1.1016 | 0.9129 | 1.3293 | 0.3128 |
| Craniectomy or Craniotomy Procedures | 1.0227 | 0.7568 | 1.3820 | 0.8839 |
| Twist Drill, Burr Hole(s), or Trephine Procedures | 1.0352 | 0.6716 | 1.5957 | 0.8753 |
| **Routine Electroencephalography (EEG) Procedures** | **1.5020** | **1.0563** | **2.1358** | **0.0235** |
| Sympathomimetics | 0.9075 | 0.7973 | 1.0329 | 0.1417 |
| Transfusion, blood or blood components | 0.8640 | 0.6809 | 1.0962 | 0.2288 |
| Emergency Department Services | 0.9453 | 0.8694 | 1.0278 | 0.1878 |
| **Disorientation, amnesia** | **1.6926** | **1.5322** | **1.8699** | **<0.0001** |
| Stupor | 0.9358 | 0.6180 | 1.4171 | 0.7541 |
| Malaise and fatigue | 0.9185 | 0.7747 | 1.0889 | 0.3274 |
| **Abnormal movements, gait disturbance** | **1.6085** | **1.4504** | **1.7839** | **<0.0001** |
| Alcohol related disorders | 1.1298 | 0.9973 | 1.2800 | 0.0552 |
| Convulsions, not elsewhere classified | 1.1275 | 0.9578 | 1.3272 | 0.1493 |
| **Epilepsy and recurrent seizures** | **1.5608** | **1.3194** | **1.8464** | **<0.0001** |

# Supplemental Table 14. GCS 3-8, Risk of Durable Cognitive Impairment by 2 Years, Controlling for Levetiracetam, Seizures, and Epilepsy

| **Covariate** | **Hazard Ratio** | **95 % CI Lower** | **95 % CI Upper** | **P > \|z\|** |
| --- | --- | --- | --- | --- |
| Gabapentin (vs. no gabapentin) | 1.1516 | 0.7105 | 1.8666 | 0.5667 |
| Levetiracetam (vs. no levetiracetam) | 1.0824 | 0.9369 | 1.2505 | 0.2822 |
| Age at Index [REF = patients 18-30 years old] |  |  |  |  |
| 30 - 35 Years | 1.0503 | 0.7887 | 1.3988 | 0.7370 |
| 35 - 40 Years | 0.8136 | 0.5725 | 1.1562 | 0.2499 |
| 40 - 45 Years | 1.0845 | 0.7833 | 1.5014 | 0.6250 |
| **45 - 50 Years** | **1.4458** | **1.0801** | **1.9354** | **0.0132** |
| 50 - 55 Years | 1.1023 | 0.8106 | 1.4990 | 0.5347 |
| 55 - 60 Years | 1.3049 | 0.9734 | 1.7492 | 0.0751 |
| **60 - 65 Years** | **1.5027** | **1.1385** | **1.9834** | **0.0040** |
| 65 - 70 Years | 1.1781 | 0.8503 | 1.6324 | 0.3245 |
| **70 - 75 Years** | **2.8247** | **2.1626** | **3.6893** | **<0.0001** |
| **75 - 80 Years** | **3.6218** | **2.7621** | **4.7491** | **<0.0001** |
| **≥80 Years** | **5.8835** | **4.5567** | **7.5966** | **<0.0001** |
| Male (vs. Female) | 1.0009 | 0.8662 | 1.1566 | 0.9902 |
| **Concussion** | **0.5408** | **0.3787** | **0.7722** | **0.0007** |
| Traumatic cerebral edema | 0.9827 | 0.8284 | 1.1657 | 0.8412 |
| **Diffuse traumatic brain injury** | **1.4164** | **1.2212** | **1.6429** | **<0.0001** |
| Epidural hemorrhage | 1.0076 | 0.7975 | 1.2731 | 0.9493 |
| **Traumatic subdural hemorrhage** | **1.3469** | **1.1499** | **1.5777** | **0.0002** |
| Traumatic subarachnoid hemorrhage | 0.9877 | 0.8422 | 1.1583 | 0.8792 |
| **Traumatic brain compression and herniation** | **0.6351** | **0.4862** | **0.8296** | **0.0009** |
| Unspecified focal traumatic brain injury | 0.8947 | 0.6949 | 1.1520 | 0.3883 |
| Contusion and laceration of right cerebrum | 1.0756 | 0.7627 | 1.5169 | 0.6777 |
| Contusion and laceration of left cerebrum | 1.2605 | 0.9089 | 1.7481 | 0.1653 |
| Traumatic hemorrhage of right cerebrum | 1.2067 | 0.9222 | 1.5788 | 0.1708 |
| Traumatic hemorrhage of left cerebrum | 0.7852 | 0.5820 | 1.0594 | 0.1136 |
| Contusion, laceration, or hemorrhage of brainstem | 0.7926 | 0.4356 | 1.4421 | 0.4466 |
| Fracture of skull and facial bones | 1.0583 | 0.9090 | 1.2322 | 0.4651 |
| Dislocation/sprain of joints/ligaments at neck level | 1.3059 | 0.9438 | 1.8068 | 0.1072 |
| Injury of nerves and spinal cord at neck level | 0.6064 | 0.4055 | 0.9068 | 0.0148 |
| Blood Pressure, Systolic (<90 mmHg vs. ≥90 mmHg) | 1.0306 | 0.8703 | 1.2205 | 0.7267 |
| Nontraumatic subarachnoid hemorrhage | 0.9451 | 0.8000 | 1.1165 | 0.5067 |
| Nontraumatic intracerebral hemorrhage | 0.9692 | 0.8053 | 1.1665 | 0.7410 |
| Unspecified nontraumatic intracranial hemorrhage | 0.9521 | 0.8114 | 1.1172 | 0.5471 |
| Cerebral infarction | 1.1322 | 0.9015 | 1.4218 | 0.2855 |
| Physiological Systems / Performance / Respiratory | 1.0067 | 0.8680 | 1.1675 | 0.9299 |
| Craniectomy or Craniotomy Procedures | 0.9156 | 0.7228 | 1.1597 | 0.4645 |
| Twist Drill, Burr Hole(s), or Trephine Procedures | 1.2579 | 0.9750 | 1.6229 | 0.0775 |
| **Routine Electroencephalography (EEG) Procedures** | **1.7813** | **1.3146** | **2.4136** | **0.0002** |
| Sympathomimetics | 0.8567 | 0.7322 | 1.0023 | 0.0535 |
| Transfusion, blood or blood components | 1.0205 | 0.8331 | 1.2501 | 0.8442 |
| Emergency Department Services | 1.0655 | 0.9313 | 1.2189 | 0.3558 |
| **Disorientation, amnesia** | **1.3014** | **1.1280** | **1.5016** | **0.0003** |
| Stupor | 0.9531 | 0.6013 | 1.5106 | 0.8379 |
| Malaise and fatigue | 1.0783 | 0.8266 | 1.4067 | 0.5782 |
| **Abnormal movements, gait disturbance** | **1.7095** | **1.4352** | **2.0363** | **<0.0001** |
| Alcohol related disorders | 1.1573 | 0.9847 | 1.3602 | 0.0762 |
| Convulsions, not elsewhere classified | 1.1602 | 0.9763 | 1.3788 | 0.0914 |
| **Epilepsy and recurrent seizures** | **1.2371** | **1.0076** | **1.5189** | **0.0421** |

# Supplemental Table 15. All GCS, Risk of Mortality by 2 Years, Controlling for Levetiracetam, Seizures, and Epilepsy

| **Covariate** | **Hazard Ratio** | **95 % CI Lower** | **95 % CI Upper** | **P > \|z\|** |
| --- | --- | --- | --- | --- |
| **Gabapentin (vs. no gabapentin)** | **0.6660** | **0.5564** | **0.7971** | **<0.0001** |
| **Levetiracetam (vs. no levetiracetam)** | **0.8266** | **0.7853** | **0.8700** | **<0.0001** |
| **GCS 13-15** | **0.3210** | **0.2798** | **0.3683** | **<0.0001** |
| **GCS 9-12** | **0.8862** | **0.7859** | **0.9992** | **0.0485** |
| **GCS 3-8** | **2.9491** | **2.5770** | **3.3750** | **<0.0001** |
| Age at Index [REF = patients 18-30 years old] |  |  |  |  |
| **30 - 35 Years** | **1.1854** | **1.0537** | **1.3336** | **0.0046** |
| **35 - 40 Years** | **1.3029** | **1.1502** | **1.4758** | **<0.0001** |
| **40 - 45 Years** | **1.2990** | **1.1410** | **1.4789** | **0.0001** |
| **45 - 50 Years** | **1.4885** | **1.3138** | **1.6865** | **<0.0001** |
| **50 - 55 Years** | **1.7097** | **1.5255** | **1.9161** | **<0.0001** |
| **55 - 60 Years** | **2.1343** | **1.9215** | **2.3707** | **<0.0001** |
| **60 - 65 Years** | **2.4389** | **2.2053** | **2.6972** | **<0.0001** |
| **65 - 70 Years** | **2.9604** | **2.6801** | **3.2700** | **<0.0001** |
| **70 - 75 Years** | **3.6236** | **3.2853** | **3.9968** | **<0.0001** |
| **75 - 80 Years** | **4.7166** | **4.2760** | **5.2027** | **<0.0001** |
| **≥80 Years** | **6.2051** | **5.6473** | **6.8181** | **<0.0001** |
| **Male (vs. Female)** | **1.1020** | **1.0482** | **1.1587** | **0.0001** |
| **Concussion** | **0.3433** | **0.3027** | **0.3895** | **<0.0001** |
| **Traumatic cerebral edema** | **1.6235** | **1.5281** | **1.7248** | **<0.0001** |
| **Diffuse traumatic brain injury** | **0.9340** | **0.8781** | **0.9935** | **0.0301** |
| **Epidural hemorrhage** | **0.7994** | **0.7248** | **0.8816** | **<0.0001** |
| **Traumatic subdural hemorrhage** | **1.0919** | **1.0340** | **1.1531** | **0.0016** |
| **Traumatic subarachnoid hemorrhage** | **0.8985** | **0.8499** | **0.9498** | **0.0002** |
| **Traumatic brain compression and herniation** | **1.2859** | **1.1890** | **1.3908** | **<0.0001** |
| Unspecified focal traumatic brain injury | 1.0614 | 0.9679 | 1.1639 | 0.2052 |
| **Contusion and laceration of right cerebrum** | **0.7977** | **0.6822** | **0.9328** | **0.0046** |
| **Contusion and laceration of left cerebrum** | **0.7283** | **0.6196** | **0.8562** | **0.0001** |
| Traumatic hemorrhage of right cerebrum | 1.0360 | 0.9350 | 1.1479 | 0.4996 |
| **Traumatic hemorrhage of left cerebrum** | **0.8689** | **0.7814** | **0.9663** | **0.0095** |
| **Contusion, laceration, or hemorrhage of brainstem** | **1.8172** | **1.5310** | **2.1567** | **<0.0001** |
| **Fracture of skull and facial bones** | **0.9271** | **0.8781** | **0.9788** | **0.0062** |
| **Dislocation/sprain of joints/ligaments at neck level** | **0.6219** | **0.5312** | **0.7281** | **<0.0001** |
| Injury of nerves and spinal cord at neck level | 0.8858 | 0.7744 | 1.0134 | 0.0773 |
| **Blood Pressure, Systolic (<90 mmHg vs. ≥90 mmHg)** | **1.4222** | **1.3404** | **1.5089** | **<0.0001** |
| **Nontraumatic subarachnoid hemorrhage** | **0.9279** | **0.8725** | **0.9869** | **0.0174** |
| **Nontraumatic intracerebral hemorrhage** | **1.3014** | **1.2190** | **1.3893** | **<0.0001** |
| **Unspecified nontraumatic intracranial hemorrhage** | **1.0954** | **1.0351** | **1.1592** | **0.0016** |
| **Cerebral infarction** | **1.1207** | **1.0303** | **1.2190** | **0.0079** |
| **Physiological Systems / Performance / Respiratory** | **1.1251** | **1.0603** | **1.1939** | **0.0001** |
| **Craniectomy or Craniotomy Procedures** | **0.7720** | **0.7074** | **0.8424** | **<0.0001** |
| Twist Drill, Burr Hole(s), or Trephine Procedures | 0.9680 | 0.8700 | 1.0771 | 0.5510 |
| **Routine Electroencephalography (EEG) Procedures** | **0.7657** | **0.6474** | **0.9057** | **0.0018** |
| **Sympathomimetics** | **1.4168** | **1.3416** | **1.4962** | **<0.0001** |
| **Transfusion, blood or blood components** | **1.5390** | **1.4408** | **1.6438** | **<0.0001** |
| **Emergency Department Services** | **0.8182** | **0.7793** | **0.8591** | **<0.0001** |
| **Disorientation, amnesia** | **0.6632** | **0.6232** | **0.7059** | **<0.0001** |
| Stupor | 0.8760 | 0.7079 | 1.0841 | 0.2235 |
| Malaise and fatigue | 1.1041 | 0.9933 | 1.2272 | 0.0663 |
| **Abnormal movements, gait disturbance** | **0.9003** | **0.8352** | **0.9704** | **0.0061** |
| **Alcohol related disorders** | **0.6146** | **0.5736** | **0.6587** | **<0.0001** |
| **Convulsions, not elsewhere classified** | **0.8113** | **0.7532** | **0.8738** | **<0.0001** |
| Epilepsy and recurrent seizures | 0.9214 | 0.8434 | 1.0066 | 0.0697 |

# Supplemental Table 16. GCS 13-15, Risk of Mortality by 2 Years, Controlling for Levetiracetam, Seizures, and Epilepsy

| **Covariate** | **Hazard Ratio** | **95 % CI Lower** | **95 % CI Upper** | **P > \|z\|** |
| --- | --- | --- | --- | --- |
| Gabapentin (vs. no gabapentin) | 0.9228 | 0.7216 | 1.1801 | 0.5220 |
| Levetiracetam (vs. no levetiracetam) | 0.9834 | 0.8826 | 1.0956 | 0.7610 |
| Age at Index [REF = patients 18-30 years old] |  |  |  |  |
| 30 - 35 Years | 1.1249 | 0.6413 | 1.9732 | 0.6814 |
| **35 - 40 Years** | **2.0921** | **1.2991** | **3.3689** | **0.0024** |
| **40 - 45 Years** | **2.4749** | **1.5584** | **3.9304** | **0.0001** |
| **45 - 50 Years** | **3.3790** | **2.2164** | **5.1515** | **<0.0001** |
| **50 - 55 Years** | **4.1816** | **2.8456** | **6.1448** | **<0.0001** |
| **55 - 60 Years** | **6.2316** | **4.3751** | **8.8757** | **<0.0001** |
| **60 - 65 Years** | **7.5889** | **5.3865** | **10.6918** | **<0.0001** |
| **65 - 70 Years** | **9.6427** | **6.8931** | **13.4891** | **<0.0001** |
| **70 - 75 Years** | **12.6752** | **9.1078** | **17.6398** | **<0.0001** |
| **75 - 80 Years** | **18.3829** | **13.2834** | **25.4400** | **<0.0001** |
| **≥80 Years** | **28.9013** | **20.9813** | **39.8107** | **<0.0001** |
| **Male (vs. Female)** | **1.3270** | **1.1999** | **1.4674** | **<0.0001** |
| **Concussion** | **0.6415** | **0.5368** | **0.7667** | **<0.0001** |
| **Traumatic cerebral edema** | **1.3871** | **1.1717** | **1.6421** | **0.0001** |
| Diffuse traumatic brain injury | 1.1193 | 0.9505 | 1.3181 | 0.1765 |
| Epidural hemorrhage | 0.8858 | 0.7046 | 1.1136 | 0.2992 |
| **Traumatic subdural hemorrhage** | **1.1928** | **1.0624** | **1.3392** | **0.0028** |
| Traumatic subarachnoid hemorrhage | 0.9990 | 0.8816 | 1.1321 | 0.9876 |
| Traumatic brain compression and herniation | 1.2385 | 0.9950 | 1.5417 | 0.0555 |
| Unspecified focal traumatic brain injury | 1.0423 | 0.8342 | 1.3021 | 0.7156 |
| Contusion and laceration of right cerebrum | 0.8098 | 0.5748 | 1.1407 | 0.2275 |
| Contusion and laceration of left cerebrum | 0.6863 | 0.4616 | 1.0204 | 0.0629 |
| Traumatic hemorrhage of right cerebrum | 1.1839 | 0.9395 | 1.4920 | 0.1525 |
| Traumatic hemorrhage of left cerebrum | 0.9391 | 0.7245 | 1.2171 | 0.6348 |
| Contusion, laceration, or hemorrhage of brainstem | 1.0164 | 0.5246 | 1.9693 | 0.9615 |
| **Fracture of skull and facial bones** | **0.8238** | **0.7299** | **0.9298** | **0.0017** |
| Dislocation/sprain of joints/ligaments at neck level | 1.0225 | 0.7323 | 1.4278 | 0.8959 |
| Injury of nerves and spinal cord at neck level | 1.2477 | 0.9275 | 1.6784 | 0.1435 |
| **Blood Pressure, Systolic (<90 mmHg vs. ≥90 mmHg)** | **1.6840** | **1.4068** | **2.0160** | **<0.0001** |
| Nontraumatic subarachnoid hemorrhage | 1.0917 | 0.9522 | 1.2516 | 0.2085 |
| **Nontraumatic intracerebral hemorrhage** | **1.2773** | **1.0981** | **1.4858** | **0.0015** |
| **Unspecified nontraumatic intracranial hemorrhage** | **1.1882** | **1.0584** | **1.3340** | **0.0035** |
| **Cerebral infarction** | **1.7159** | **1.4296** | **2.0594** | **<0.0001** |
| **Physiological Systems / Performance / Respiratory** | **2.9590** | **2.5387** | **3.4489** | **<0.0001** |
| Craniectomy or Craniotomy Procedures | 1.2977 | 0.9984 | 1.6868 | 0.0514 |
| Twist Drill, Burr Hole(s), or Trephine Procedures | 1.0680 | 0.7198 | 1.5846 | 0.7439 |
| Routine Electroencephalography (EEG) Procedures | 0.7456 | 0.4689 | 1.1856 | 0.2148 |
| Sympathomimetics | 0.9221 | 0.8051 | 1.0560 | 0.2410 |
| **Transfusion, blood or blood components** | **1.6091** | **1.3307** | **1.9457** | **<0.0001** |
| **Emergency Department Services** | **0.6562** | **0.5949** | **0.7239** | **<0.0001** |
| Disorientation, amnesia | 1.1096 | 0.9806 | 1.2556 | 0.0991 |
| Stupor | 0.7691 | 0.4596 | 1.2870 | 0.3175 |
| **Malaise and fatigue** | **1.4182** | **1.1935** | **1.6852** | **0.0001** |
| **Abnormal movements, gait disturbance** | **1.2359** | **1.0898** | **1.4016** | **0.0010** |
| Alcohol related disorders | 1.1291 | 0.9841 | 1.2955 | 0.0834 |
| **Convulsions, not elsewhere classified** | **1.3302** | **1.1268** | **1.5702** | **0.0007** |
| **Epilepsy and recurrent seizures** | **1.3929** | **1.1649** | **1.6656** | **0.0003** |

# Supplemental Table 17. GCS 3-8, Risk of Mortality by 2 Years, Controlling for Levetiracetam, Seizures, and Epilepsy

| **Covariate** | **Hazard Ratio** | **95 % CI Lower** | **95 % CI Upper** | **P > \|z\|** |
| --- | --- | --- | --- | --- |
| **Gabapentin (vs. no gabapentin)** | **0.5391** | **0.4066** | **0.7147** | **<0.0001** |
| **Levetiracetam (vs. no levetiracetam)** | **0.7729** | **0.7280** | **0.8205** | **<0.0001** |
| Age at Index [REF = patients 18-30 years old] |  |  |  |  |
| **30 - 35 Years** | **1.1655** | **1.0322** | **1.3161** | **0.0135** |
| **35 - 40 Years** | **1.2914** | **1.1326** | **1.4725** | **0.0001** |
| **40 - 45 Years** | **1.2600** | **1.0978** | **1.4461** | **0.0010** |
| **45 - 50 Years** | **1.3967** | **1.2199** | **1.5992** | **<0.0001** |
| **50 - 55 Years** | **1.5546** | **1.3738** | **1.7593** | **<0.0001** |
| **55 - 60 Years** | **1.7982** | **1.5996** | **2.0216** | **<0.0001** |
| **60 - 65 Years** | **2.0482** | **1.8297** | **2.2928** | **<0.0001** |
| **65 - 70 Years** | **2.3980** | **2.1410** | **2.6858** | **<0.0001** |
| **70 - 75 Years** | **2.7442** | **2.4478** | **3.0765** | **<0.0001** |
| **75 - 80 Years** | **3.2743** | **2.9060** | **3.6892** | **<0.0001** |
| **≥80 Years** | **3.7002** | **3.2863** | **4.1663** | **<0.0001** |
| Male (vs. Female) | 1.0150 | 0.9558 | 1.0779 | 0.6275 |
| **Concussion** | **0.2561** | **0.2073** | **0.3163** | **<0.0001** |
| **Traumatic cerebral edema** | **1.5684** | **1.4674** | **1.6764** | **<0.0001** |
| **Diffuse traumatic brain injury** | **0.8967** | **0.8379** | **0.9596** | **0.0016** |
| **Epidural hemorrhage** | **0.7489** | **0.6693** | **0.8380** | **<0.0001** |
| Traumatic subdural hemorrhage | 1.0366 | 0.9725 | 1.1049 | 0.2695 |
| **Traumatic subarachnoid hemorrhage** | **0.8481** | **0.7953** | **0.9044** | **<0.0001** |
| **Traumatic brain compression and herniation** | **1.2977** | **1.1907** | **1.4144** | **<0.0001** |
| Unspecified focal traumatic brain injury | 1.0842 | 0.9768 | 1.2034 | 0.1288 |
| Contusion and laceration of right cerebrum | 0.8379 | 0.6994 | 1.0039 | 0.0551 |
| **Contusion and laceration of left cerebrum** | **0.6974** | **0.5754** | **0.8452** | **0.0002** |
| Traumatic hemorrhage of right cerebrum | 0.9818 | 0.8699 | 1.1082 | 0.7667 |
| **Traumatic hemorrhage of left cerebrum** | **0.8347** | **0.7386** | **0.9432** | **0.0038** |
| **Contusion, laceration, or hemorrhage of brainstem** | **1.9108** | **1.5950** | **2.2891** | **<0.0001** |
| **Fracture of skull and facial bones** | **0.9143** | **0.8584** | **0.9738** | **0.0054** |
| **Dislocation/sprain of joints/ligaments at neck level** | **0.5865** | **0.4880** | **0.7049** | **<0.0001** |
| **Injury of nerves and spinal cord at neck level** | **0.7823** | **0.6688** | **0.9151** | **0.0022** |
| **Blood Pressure, Systolic (<90 mmHg vs. ≥90 mmHg)** | **1.3995** | **1.3125** | **1.4923** | **<0.0001** |
| **Nontraumatic subarachnoid hemorrhage** | **0.9081** | **0.8444** | **0.9767** | **0.0094** |
| **Nontraumatic intracerebral hemorrhage** | **1.2534** | **1.1621** | **1.3520** | **<0.0001** |
| Unspecified nontraumatic intracranial hemorrhage | 1.0401 | 0.9717 | 1.1133 | 0.2574 |
| Cerebral infarction | 1.0027 | 0.9085 | 1.1067 | 0.9569 |
| Physiological Systems / Performance / Respiratory | 0.9702 | 0.9116 | 1.0327 | 0.3422 |
| **Craniectomy or Craniotomy Procedures** | **0.7853** | **0.7138** | **0.8639** | **<0.0001** |
| **Twist Drill, Burr Hole(s), or Trephine Procedures** | **0.9635** | **0.8608** | **1.0784** | **0.5173** |
| **Routine Electroencephalography (EEG) Procedures** | **0.7241** | **0.5986** | **0.8758** | **0.0009** |
| **Sympathomimetics** | **1.5209** | **1.4289** | **1.6187** | **<0.0001** |
| **Transfusion, blood or blood components** | **1.4193** | **1.3203** | **1.5256** | **<0.0001** |
| **Emergency Department Services** | **0.9221** | **0.8700** | **0.9774** | **0.0063** |
| **Disorientation, amnesia** | **0.5440** | **0.5037** | **0.5874** | **<0.0001** |
| Stupor | 0.8704 | 0.6777 | 1.1179 | 0.2771 |
| Malaise and fatigue | 0.9041 | 0.7823 | 1.0449 | 0.1722 |
| **Abnormal movements, gait disturbance** | **0.7106** | **0.6407** | **0.7880** | **<0.0001** |
| **Alcohol related disorders** | **0.5252** | **0.4832** | **0.5709** | **<0.0001** |
| **Convulsions, not elsewhere classified** | **0.7128** | **0.6530** | **0.7780** | **<0.0001** |
| **Epilepsy and recurrent seizures** | **0.8307** | **0.7463** | **0.9247** | **0.0007** |

# Supplemental Table 18. Criteria for Moderate TBI

Formally, we determined that GCS 13-15 indicated a mild TBI, GCS 9-12 indicated a moderate TBI, and GCS 3-8 indicated a severe TBI. However, very few patients had a recorded moderate TBI, so a hybrid definition ws created as a sensitivity analysis. Patients were included if they had at least one inclusion criteria; patients were excluded if they had at least one exclusion criteria.

For inclusion criteria, we reasoned that a deficit in at least one category would indicate a moderate-level TBI if a patient’s overall GCS was neither 3-8 nor 13-15. Further, we reasoned that if a patient had “no response” in a given category, their injury was likely severe. For these reasons, our hybrid definition inclusion criteria included sub-optimal responses.

For exclusion criteria, we removed patients with GCS 13-15 and GCS 3-8 as these met formal criteria for mild and severe TBI, respectively. We removed patients with paralytic strabismus and “sequelae of cerebrovascular disease” as these categories include physical exam findings suggestive of severe injury. Patients requiring ventriculostomy, ventricular bypass, other surgical interventions, and mechanical ventilation were also likely to be severely obtunded, warranting a label of severe TBI. Last, we excluded patients with “no response” in any GCS category as described in the inclusion criteria.

| **Inclusion Criteria** | | **Exclusion Criteria** | |
| --- | --- | --- | --- |
| **Code** | **Description** | **Code** | **Description** |
| R40.212 | Coma scale, eyes open, to pain | R40.243 | GCS 3-8 |
| R40.213 | Coma scale, eyes open, to sound | R40.241 | GCS 13-15 |
| R40.222 | Coma scale, best verbal response, incomprehensible words | H49 | Paralytic strabismus |
| R40.223 | Coma scale, best verbal response, inappropriate words | I69 | Sequelae of cerebrovascular disease |
| R40.224 | Coma scale, best verbal response, confused conversation | 02.2 | Ventriculostomy |
| R40.232 | Coma scale, best motor response, extension | 5A19 | Mechanical ventilation |
| R40.233 | Coma scale, best motor response, abnormal flexion | 0016 | Ventricular bypass |
| R40.234 | Coma scale, best motor response, flexion withdrawal | 1009069 | Surgical procedures on the skull, meninges, and brain |
| R40.235 | Coma scale, best motor response, localizes pain | R40.211 | Coma scale, eyes open, never |
| R40.242 | GCS 9-12 | R40.221 | Coma scale, best verbal response, none |
|  |  | R40.231 | Coma scale, best motor response, none |

# Supplemental Table 19. Risk of Durable Cognitive Impairment by 2 Years in Moderate TBI

| **Covariate** | **Hazard Ratio** | **95 % CI Lower** | **95 % CI Upper** | **P > \|z\|** |
| --- | --- | --- | --- | --- |
| Gabapentin (vs. no gabapentin) | 0.8203 | 0.6565 | 1.0250 | 0.0814 |
| Age at Index [REF = patients 18-30 years old] |  |  |  |  |
| 30 - 35 Years | 1.0899 | 0.6190 | 1.9189 | 0.7655 |
| 35 - 40 Years | 1.1359 | 0.6372 | 2.0250 | 0.6657 |
| 40 - 45 Years | 0.9410 | 0.4943 | 1.7914 | 0.8531 |
| 45 - 50 Years | 1.4080 | 0.8084 | 2.4524 | 0.2267 |
| **50 - 55 Years** | **2.6998** | **1.7242** | **4.2276** | **<0.0001** |
| **55 - 60 Years** | **3.4889** | **2.3248** | **5.2358** | **<0.0001** |
| **60 - 65 Years** | **4.4138** | **2.9942** | **6.5065** | **<0.0001** |
| **65 - 70 Years** | **6.3143** | **4.3889** | **9.0845** | **<0.0001** |
| **70 - 75 Years** | **12.8846** | **9.1834** | **18.0777** | **<0.0001** |
| **75 - 80 Years** | **19.4326** | **13.9340** | **27.1011** | **<0.0001** |
| **≥80 Years** | **29.8114** | **21.5429** | **41.2535** | **<0.0001** |
| **Male (vs. Female)** | **0.8769** | **0.7915** | **0.9716** | **0.0120** |
| **Concussion** | **0.7456** | **0.6149** | **0.9041** | **0.0028** |
| Traumatic cerebral edema | 0.9951 | 0.8236 | 1.2025 | 0.9597 |
| Diffuse traumatic brain injury | 1.2035 | 1.0129 | 1.4299 | 0.0352 |
| Epidural hemorrhage | 1.0635 | 0.7879 | 1.4355 | 0.6875 |
| **Traumatic subdural hemorrhage** | **1.2147** | **1.0787** | **1.3680** | **0.0013** |
| Traumatic subarachnoid hemorrhage | 0.9702 | 0.8569 | 1.0984 | 0.6325 |
| **Traumatic brain compression and herniation** | **0.6913** | **0.5389** | **0.8869** | **0.0037** |
| Unspecified focal traumatic brain injury | 1.0173 | 0.7867 | 1.3155 | 0.8959 |
| Contusion and laceration of right cerebrum | 0.8304 | 0.5915 | 1.1657 | 0.2829 |
| Contusion and laceration of left cerebrum | 1.1292 | 0.8201 | 1.5548 | 0.4565 |
| Traumatic hemorrhage of right cerebrum | 1.1693 | 0.9201 | 1.4861 | 0.2009 |
| Traumatic hemorrhage of left cerebrum | 0.8836 | 0.6988 | 1.1173 | 0.3014 |
| Contusion, laceration, or hemorrhage of brainstem | 1.2484 | 0.6200 | 2.5138 | 0.5343 |
| **Fracture of skull and facial bones** | **0.8539** | **0.7578** | **0.9622** | **0.0095** |
| Dislocation/sprain of joints/ligaments at neck level | 1.3843 | 0.9162 | 2.0913 | 0.1225 |
| Injury of nerves and spinal cord at neck level | 0.9628 | 0.5967 | 1.5535 | 0.8766 |
| Blood Pressure, Systolic (<90 mmHg vs. ≥90 mmHg) | 1.0996 | 0.8714 | 1.3876 | 0.4238 |
| Nontraumatic subarachnoid hemorrhage | 0.9629 | 0.8407 | 1.1028 | 0.5851 |
| Nontraumatic intracerebral hemorrhage | 0.9938 | 0.8486 | 1.1639 | 0.9389 |
| Unspecified nontraumatic intracranial hemorrhage | 1.0525 | 0.9324 | 1.1882 | 0.4079 |
| Cerebral infarction | 0.9752 | 0.7694 | 1.2360 | 0.8356 |
| Routine Electroencephalography (EEG) Procedures | 1.1107 | 0.7705 | 1.6010 | 0.5738 |
| **Sympathomimetics** | **0.8288** | **0.7125** | **0.9641** | **0.0150** |
| Transfusion, blood or blood components | 0.7871 | 0.6205 | 0.9984 | 0.0485 |
| Emergency Department Services | 1.0392 | 0.9374 | 1.1521 | 0.4648 |
| **Disorientation, amnesia** | **1.1228** | **1.0064** | **1.2527** | **0.0380** |
| Stupor | 0.8988 | 0.5179 | 1.5598 | 0.7045 |
| Malaise and fatigue | 0.9940 | 0.8249 | 1.1977 | 0.9493 |
| **Abnormal movements, gait disturbance** | **1.4865** | **1.3260** | **1.6663** | **<0.0001** |
| **Alcohol related disorders** | **0.7950** | **0.6767** | **0.9339** | **0.0052** |

# Supplemental Table 20. Risk of Mortality by 2 Years in Moderate TBI

| **Covariate** | **Hazard Ratio** | **95 % CI Lower** | **95 % CI Upper** | **P > \|z\|** |
| --- | --- | --- | --- | --- |
| **Gabapentin (vs. no gabapentin)** | **0.5996** | **0.4073** | **0.8827** | **0.0095** |
| Age at Index [REF = patients 18-30 years old] |  |  |  |  |
| 30 - 35 Years | 1.8987 | 0.6584 | 5.4751 | 0.2354 |
| 35 - 40 Years | 2.1085 | 0.7312 | 6.0802 | 0.1674 |
| **40 - 45 Years** | **4.8238** | **1.9972** | **11.6510** | **0.0005** |
| 45 - 50 Years | 1.9112 | 0.6247 | 5.8473 | 0.2563 |
| **50 - 55 Years** | **4.7032** | **1.9468** | **11.3625** | **0.0006** |
| **55 - 60 Years** | **8.0360** | **3.6544** | **17.6709** | **<0.0001** |
| **60 - 65 Years** | **9.2815** | **4.2659** | **20.1943** | **<0.0001** |
| **65 - 70 Years** | **18.2482** | **8.7149** | **38.2102** | **<0.0001** |
| **70 - 75 Years** | **27.5532** | **13.3352** | **56.9306** | **<0.0001** |
| **75 - 80 Years** | **36.5774** | **17.7989** | **75.1678** | **<0.0001** |
| **≥80 Years** | **67.6337** | **33.1868** | **137.8353** | **<0.0001** |
| **Male (vs. Female)** | **1.3098** | **1.1246** | **1.5255** | **0.0005** |
| Concussion | 1.2246 | 0.9563 | 1.5683 | 0.1083 |
| Traumatic cerebral edema | 1.0845 | 0.8436 | 1.3940 | 0.5269 |
| Diffuse traumatic brain injury | 1.3763 | 0.9368 | 2.0220 | 0.1037 |
| Epidural hemorrhage | 1.1317 | 0.9497 | 1.3486 | 0.1668 |
| Traumatic subdural hemorrhage | 0.8662 | 0.7220 | 1.0392 | 0.1220 |
| **Traumatic subarachnoid hemorrhage** | **1.5209** | **1.1607** | **1.9929** | **0.0024** |
| Traumatic brain compression and herniation | 1.1800 | 0.8540 | 1.6306 | 0.3157 |
| **Unspecified focal traumatic brain injury** | **0.4882** | **0.2820** | **0.8452** | **0.0105** |
| **Contusion and laceration of right cerebrum** | **1.6542** | **1.1212** | **2.4405** | **0.0112** |
| Contusion and laceration of left cerebrum | 0.9226 | 0.6464 | 1.3169 | 0.6573 |
| Traumatic hemorrhage of right cerebrum | 1.2997 | 0.9709 | 1.7398 | 0.0781 |
| Traumatic hemorrhage of left cerebrum | 1.4806 | 0.6064 | 3.6151 | 0.3888 |
| Contusion, laceration, or hemorrhage of brainstem | 0.9199 | 0.7735 | 1.0939 | 0.3450 |
| **Fracture of skull and facial bones** | **1.9132** | **1.1049** | **3.3127** | **0.0205** |
| Dislocation/sprain of joints/ligaments at neck level | 0.9333 | 0.4851 | 1.7955 | 0.8362 |
| Injury of nerves and spinal cord at neck level | 1.2246 | 0.9563 | 1.5683 | 0.1083 |
| **Blood Pressure, Systolic (<90 mmHg vs. ≥90 mmHg)** | **2.0739** | **1.5934** | **2.6993** | **<0.0001** |
| Nontraumatic subarachnoid hemorrhage | 0.9994 | 0.8191 | 1.2193 | 0.9950 |
| **Nontraumatic intracerebral hemorrhage** | **1.5579** | **1.2771** | **1.9006** | **<0.0001** |
| **Unspecified nontraumatic intracranial hemorrhage** | **1.2007** | **1.0111** | **1.4259** | **0.0370** |
| **Cerebral infarction** | **1.8570** | **1.4228** | **2.4237** | **<0.0001** |
| Routine Electroencephalography (EEG) Procedures | 1.3945 | 0.8521 | 2.2820 | 0.1858 |
| Sympathomimetics | 0.8121 | 0.6511 | 1.0130 | 0.0649 |
| **Transfusion, blood or blood components** | **1.5396** | **1.1763** | **2.0152** | **0.0017** |
| **Emergency Department Services** | **0.7726** | **0.6653** | **0.8972** | **0.0007** |
| Disorientation, amnesia | 0.9493 | 0.8054 | 1.1188 | 0.5344 |
| Stupor | 1.0384 | 0.4889 | 2.2054 | 0.9219 |
| Malaise and fatigue | 1.1282 | 0.8717 | 1.4601 | 0.3594 |
| **Abnormal movements, gait disturbance** | **1.1969** | **1.0112** | **1.4168** | **0.0367** |
| **Alcohol related disorders** | **0.7196** | **0.5655** | **0.9156** | **0.0074** |
